# Addressing the properties of “Metallo-DNA” with a Ag(i)-mediated supramolecular duplex†
†Electronic supplementary information (ESI) available: Experimental details of the sample preparation, characterisation and data analysis (AFM image processing). CCDC 1861420. For ESI and crystallographic data in CIF or other electronic format see DOI: 10.1039/c8sc05103h


**DOI:** 10.1039/c8sc05103h

**Published:** 2019-02-08

**Authors:** Liam Mistry, Osama El-Zubir, Gema Dura, William Clegg, Paul G. Waddell, Thomas Pope, Werner A. Hofer, Nick G. Wright, Benjamin R. Horrocks, Andrew Houlton

**Affiliations:** a Chemical Nanoscience Laboratory , School of Natural & Environmental Sciences , Newcastle University , Newcastle upon Tyne , NE1 7RU , UK . Email: Andrew.houlton@ncl.ac.uk; b Chemistry , School of Natural & Environmental Sciences , Newcastle University , Newcastle upon Tyne , NE1 7RU , UK; c School of Engineering , Newcastle University , Newcastle upon Tyne , NE1 7RU , UK

## Abstract

The silver-nucleoside complex [Ag(i)-(*N*3-cytidine)_2_], **1**, self-assembles to form a supramolecular metal-mediated base-pair array highly analogous to those seen in metallo-DNA. A combination of complementary hydrogen-bonding, hydrophobic and argentophilic interactions drive the formation of a double-helix with a continuous silver core. Electrical measurements on **1** show that despite having Ag···Ag distances within <5% of the metallic radii, the material is electrically insulating. This is due to the electronic structure which features a filled valence band, an empty conduction band dominated by the ligand, and a band gap of 2.5 eV. Hence, as-prepared, such Ag(i)-DNA systems should not be considered molecular nanowires but, at best, proto-wires. The structural features seen in **1** are essentially retained in the corresponding organogel which exhibits thixotropic self-healing that can be attributed to the reversible nature of the intermolecular interactions. Photo-reduced samples of the gel exhibit luminescence confirming that these poly-cytidine sequences appropriately pre-configure silver ions for the formation of quantum-confined metal clusters in line with contemporary views on DNA-templated clusters. Microscopy data reveals the resulting metal cluster/particles are approximately spherical and crystalline with lattice spacing (111) similar to bulk Ag.

## Abstract 2

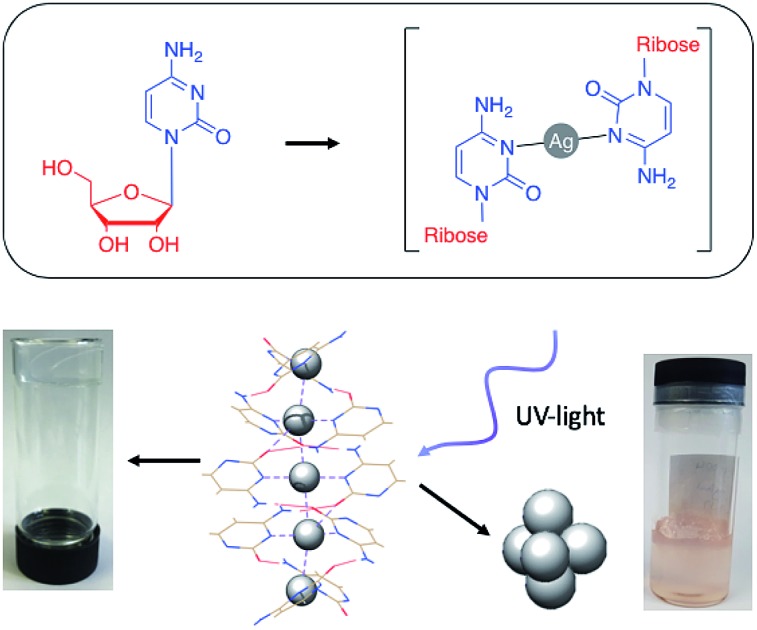
The silver-nucleoside complex [Ag(i)-(*N*3-cytidine)_2_]^+^, **1**, self-assembles to form a supramolecular metal-mediated base-pair array highly analogous to those seen in metallo-DNA.

## Introduction

**Scheme 1:**
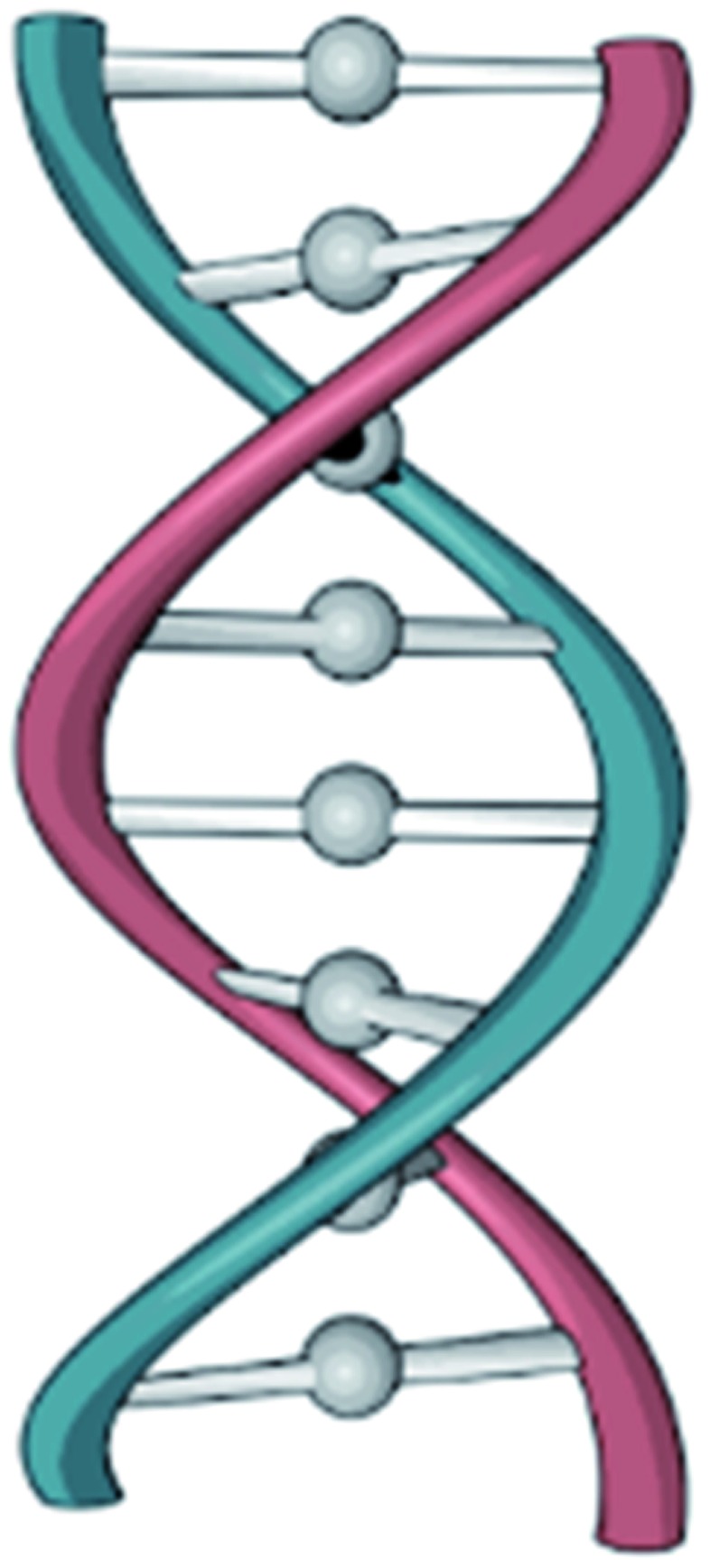
An illustration of metal-mediated DNA duplex.

Metallo-DNA, that is double-stranded DNA containing metal-mediated base pairs, is an important class of nano-materials that seeks to combine the programmability of DNA with the unique properties of metals.^1^–^7^ Essentially, the hydrogen bonds between base pairs of natural DNA are replaced by coordinate bonds resulting in a metallic core running through the double helix (Scheme 1). This has proven to be a powerful method for controlling the assembly of metal ions into linear arrays and both homo- and heterometallic systems have been demonstrated. However, for most metal ions artificial nucleosides are required to accommodate the various demands of metal ion coordination.^5^,^6^,^8^,^9^

**Scheme 2:**
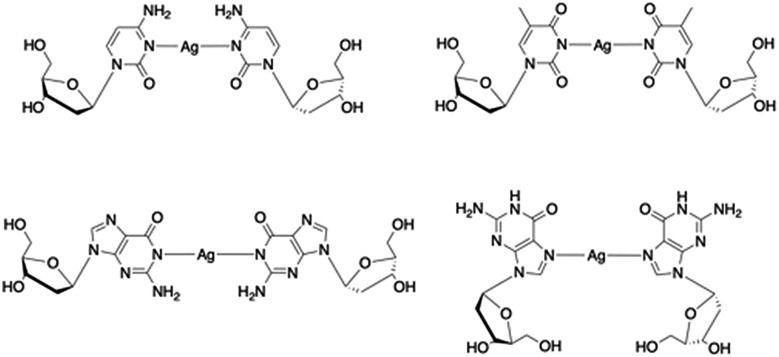
Examples of crystallographically characterised Ag(i)-mediated base pairs with natural nucleosides identified in metallo-DNA.

Silver ions are an exception to this as they can form the necessary metal-mediated base pairs with natural nucleosides.^7^,^10^–^18^ Consequently, the combination of silver chemistry with DNA-based structure building is particularly promising for the synthesis of nanomaterials, *e.g.* nanowires,^19^–^23^ quantum-confined metal clusters^24^–^31^ and plasmonic materials^32^,^33^ as well as the development of a bottom-up approach to functional molecular systems. While most common with cytidine, other silver-mediated pairings including Watson–Crick purine–pyrimidine combinations are possible (Scheme 2).^12^,^18^,^22^,^34^ Examples have been identified in crystal structures of duplex oligonucleotides^12^,^18^ including that of a dodecamer d(GGACTC^Br^GATCC).^22^ In this last example all the base pairs are metal-mediated and generate an infinite chain of Ag ions running through the crystal structure. Analogous arrays of Ag(i) ions have recently been shown to be a feature in the solid state structure of simple nucleobase complexes of the form [Ag(i)-(*N*3-alkyl-cytosine)_2_]^+^ isolated from organic solvent.^35^,^36^ Such metallo-DNA systems are frequently considered as molecular or nano-wires^22^,^37^,^38^ on account of the contiguous linear array of metal ions produced. However, studies on their electrical properties are rather scant^39^–^42^ and only one, reported while our manuscript was under review, is for a crystallographically characterised material.^36^

Here, we report the metal-mediated nucleoside complex, [Ag(i)-(*N*3-cytidine)_2_], **1** that self-assembles to yield a metallo-DNA analogue in both solid and gel states. This compound provides a convincing model of a metallo-DNA duplex, containing, as it does, both the nucleobase and ribose moieties, allowing the physico-chemical properties of such systems to be examined. Here we address their putative nanowire status and provide insight into the reduction behaviour of this type of system with the formation of luminescent quantum-confined metal clusters. We add that while our work was under review a study by Galindo *et al.^36^* that includes examination of the electrical properties and cold-H plasma reduction of the nucleobase complex [Ag(i)-(methylcytosine)_2_]^+^ appeared. Our work here is complementary to these results and usefully extends it by full *I*–*V* characterisation on oriented single crystals, scanned conductance (SCM/EFM) measurements on molecular-scale fibres and calculation of the local density of states. Furthermore, we show for the first time that this type of self-assembled metallo-array exhibits self-healing properties when in the gel form, a feature which can be rationalised from the reversibility of the inter-complex hydrogen- and argentophilic-bonding in the supramolecular structure.

## Results and discussion

### Synthesis and characterisation of a “metallo-DNA” model [Ag(*N*3-cytidine)_2_][NO_3_], **1**

**Fig. 1:**
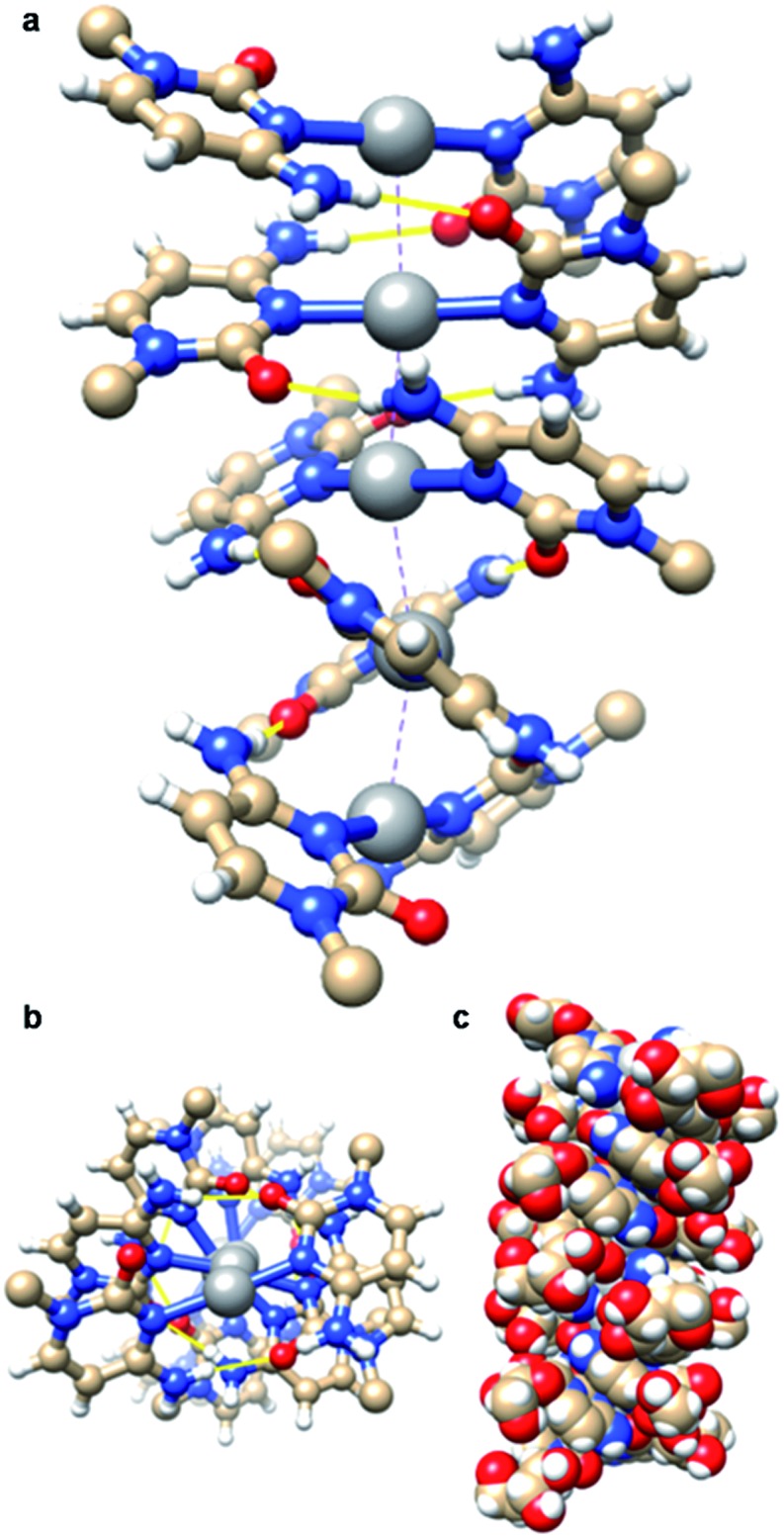
The continuous linear array of silver ions in the right-handed supramolecular double helix of metallo-DNA model [Ag(i)-(*N*3-cytidine)_2_]^+^, **1**. View (a) onto and (b) down the helix axis of a section of the contiguous 1D supramolecular silver array. Section corresponds to the five independent molecules spanning the crystallographic *c*-axis of the unit cell. Inter-nucleoside hydrogen bonding is indicated as yellow lines and the ribose units are removed for clarity. (c) Space-filling model containing ten complexes (two pitches of the double helix) complete with ribose groups.

The aqueous reaction between AgNO_3_ and cytidine in a 1 : 2 stoichiometric ratio yielded [Ag(*N*3-cytidine)_2_][NO_3_], **1**, as a white powder in quantitative yield. Recrystallisation from water/acetonitrile gave colourless needle-shaped crystals. X-ray diffraction analysis revealed **1** to crystallise in the chiral space group *P*3_2_ with five crystallographically independent [Ag(i)(*N*3-cytidine)_2_]^+^ complex ions, accounting for the relatively large unit cell (*a* = *b* = 30.382(7) Å; *c* = 14.976(3) Å). These complex cations assemble through a combination of complementary hydrogen bonds and argentophilic interactions to form a right-handed supramolecular double helix extending continuously through the crystal structure (Fig. 1). The resulting silver ion array forms the core of the duplex with the ∼15 Å crystallographic *c*-axis defining the helical pitch. The chain of Ag atoms is almost linear, with deviations of 0.162–0.273 Å, from the mean straight line which is parallel to the trigonal *c*-axis, and with Ag···Ag···Ag angles in the range 162.34(7)–170.63(6)°. The diameter of the duplex, as measured between 5′-OH groups on individual complex ions, is similar to that of natural DNA duplex at 17.5(1) Å. The pyrimidine rings of each metal complex are twisted significantly from co-planarity (inter-planar dihedral angle range; 71.2(5)–74.9(5)°) and adopt a slight cisoid conformation. For each “strand” of the duplex, this conformation generates a polar arrangement of nucleosides and directs the formation of complementary NH···O=C hydrogen bonds between neighbouring complexes. Each individual complex forms two pairs of NH···O hydrogen bonds to neighbours (range of O2···N4: 2.870(18)–3.020(17) Å). A similar pattern of inter base-pair hydrogen bonding between silver-mediated base pairs is observed by Kondo *et al.*^22^ in a metallo-DNA [(Ag_11_-(dodecacamer)_2_)] and in the organo-soluble complex [Ag(i)–*N*3-(*N*1-hexylcytosine)_2_].^35^ However, in **1** the argentophilic interactions are stronger as indicated by the shorter Ag···Ag distances in the range 2.982(2)–3.055(2) Å, close to the metallic radius of 2.88 Å. For comparison, the intermetallic distances in [(Ag_11_-(dodecacamer)_2_)] are 3.2–3.4 Å; and 3.162–3.235 Å in [Ag(i)–*N*3-(*N*1-hexylcytosine)_2_].^35^ Interestingly, the recently reported [Ag(1-Me-cytosine)_2_]^+^ complex features similarly short Ag···Ag distances (2.902–3.088(2) Å) as **1**.^36^ This finding indicates that the ribose moiety of the nucleoside does not impose additional steric hinderance to inter-complex interactions allowing short argentophilic bonding.

### Self-healing metallo-DNA gel formation

**Fig. 2:**
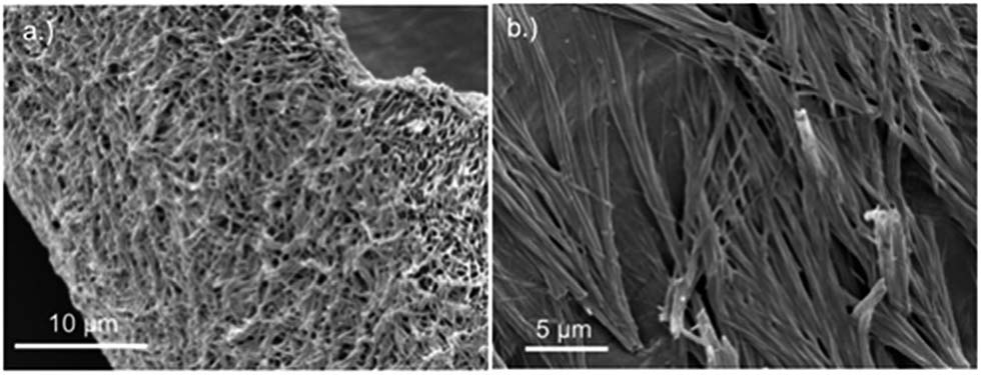
SEM images of bis(cytidine)-silver(i) xerogel, **1_MeOH_**. (a) Xerogel showing the entangled porous polymer matrix. (b) Micrograph showing bundling of fibres.

**Fig. 3:**
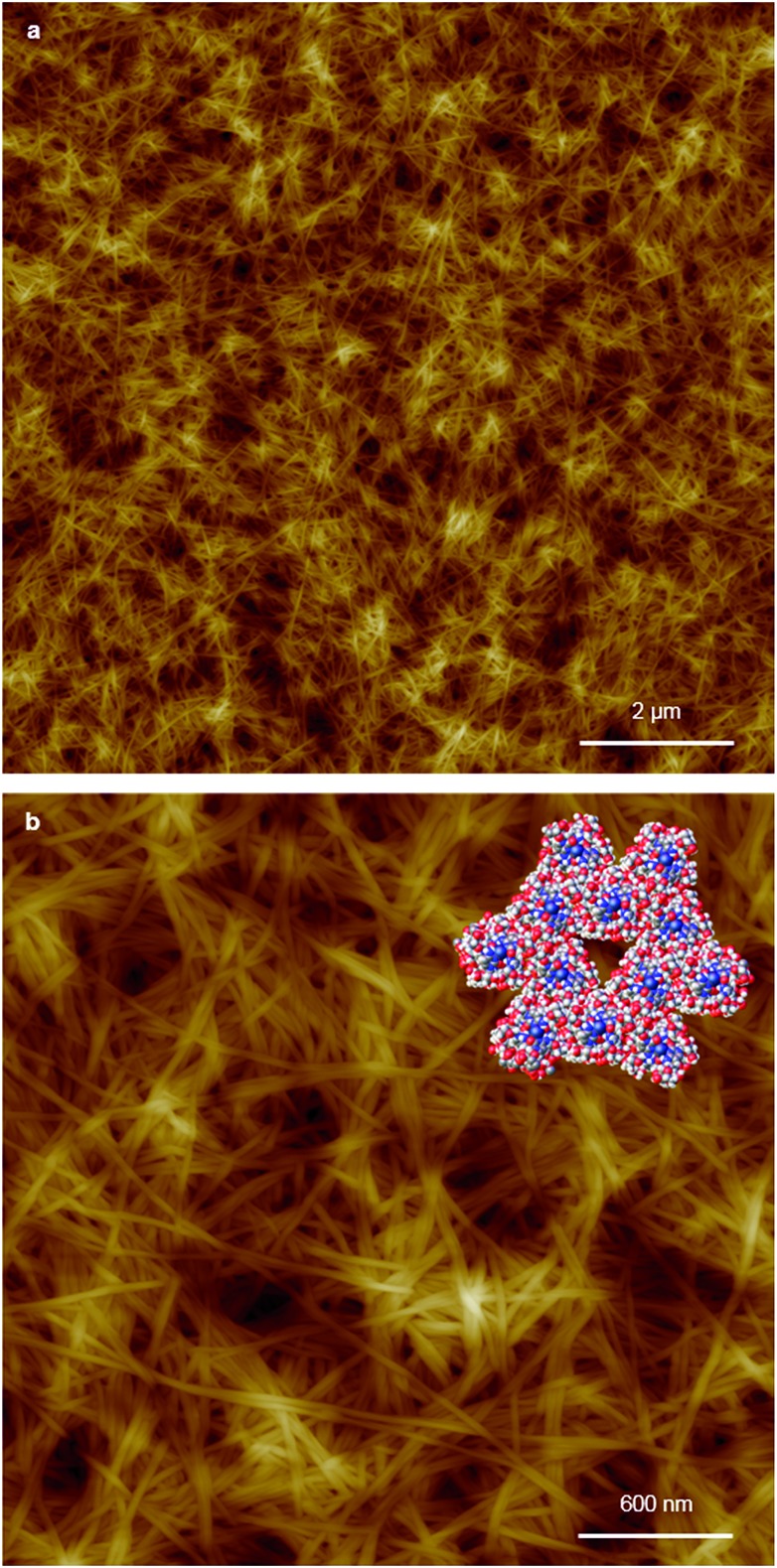
AFM images of xerogel **Xe1** on a silicon wafer. Large-scan (a) and a zoom area (b) of AFM height images illustrating the regular nature of the fibres and (inset) space-filling molecular model derived from **1** of a twelve duplex “fibre” with corresponding, 6.3 nm diameter of individual fibres.

Replacing water with methanol in the above reaction gave a colourless sample-spanning gel-like material, **1_MeOH_**. ES-MS and FTIR indicate the formation of the expected bis-cytidine complex (ESI, Fig. S3 and S6†). Characterisation by SEM (Fig. 2, ESI, Fig. S8†) reveals one-dimensional fibres, consistent with the extended supramolecular structure of **1**. These are many microns in length and entangle to form a porous network for solvent trapping (Fig. 2a) and bundling of fibres is seen (Fig. 2b). Tapping-mode AFM shows that at the as-prepared concentration (4 mg ml^–1^) the fibres are remarkably regular in diameter and individual fibres exhibit little tendency to coil (Fig. 3). The rather monodisperse nature indicates a regular hierarchical assembly process and image analysis establishes these individual fibres to be approx. 6.3 ± 0.45 nm in diameter (ESI, Fig. S9–S11†). Assuming a structure based on **1** such an assembly would contain twelve individual Ag-duplexes (Fig. 3b, inset).

**Fig. 4:**
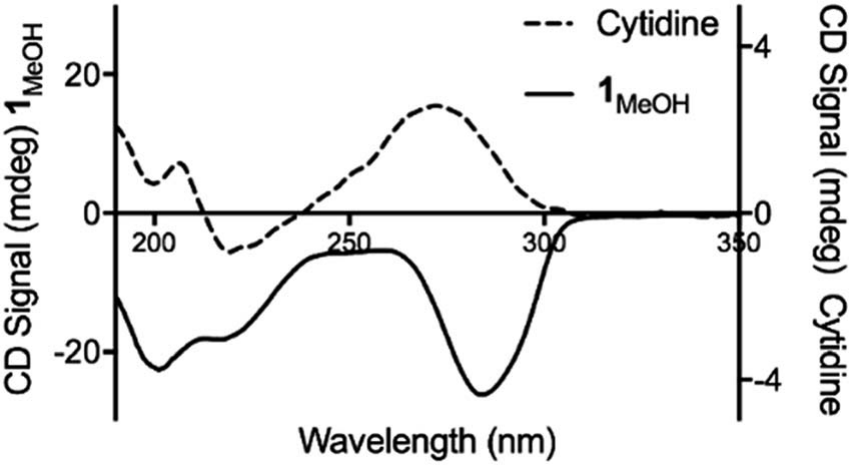
Circular dichroism spectra for cytidine (dotted line) and **1_MeOH_** (solid line) at concentrations of 4 mg ml^–1^.

Consistent with this, the gel displays markedly enhanced chiroptic absorptions compared to the parent nucleoside that differ in the sign of the main ∼280 nm absorption band (Fig. 4). The observed profile is diagnostic of helical arrangements of Ag-mediated cytidine base pair sequences^18^,^43^–^45^ which are structurally analogous to the arrangement in **1**.

**Fig. 5:**
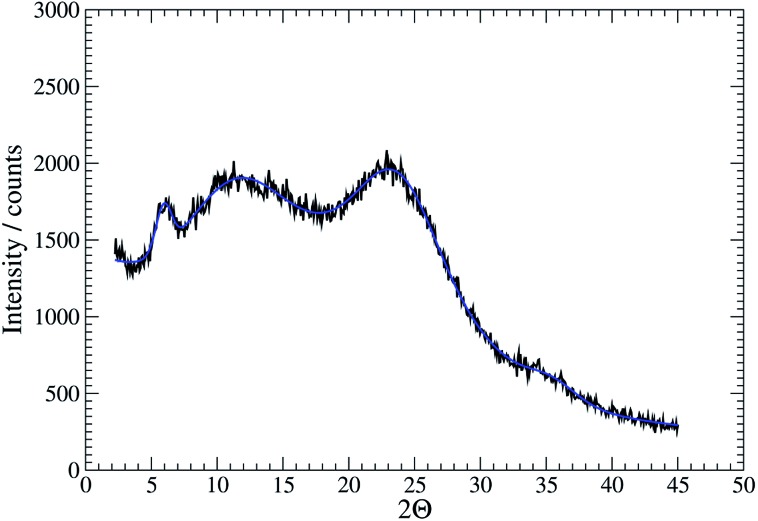
XRD data for the gel **1_MeOH_** with fit shown in blue.

Further confirmation of the isostructural nature was provided by analysis of XRD patterns. For the gel **1_MeOH_** these show rather broad features typical of polymeric materials (Fig. 5). This is expected given the fibrous nature of the gel observed in AFM. Nevertheless, the data can be analysed by fitting to a sum of Gaussian functions in a similar manner to the Rietveld method. This analysis shows a relatively narrow peak near 2*θ* = 5.94° (14.8 Å) which can be interpreted as the broadened 001 reflection corresponding to the *c*-axis of the unit cell observed in the single crystal data. This data suggests the structure of the gel fibres is essentially the same as the crystal structure of **1** with the axis of the fibres lying along the *c*-axis of the crystal structure. When the gel is freeze-dried or the solvent simply allowed to evaporate in air, the XRD pattern for the resulting xerogel **Xe1** shows sharper features that indicate a greater degree of crystalline order, but the major peak at 2*θ* = 6.43° corresponds to a distance of 13.7 Å (ESI, Fig. S12†). If a microcrystalline powder of **1** is also freeze-dried a similar reflection is also observed alongside the reflection corresponding to *c*-axis in the single crystal data (ESI, Fig. S12†). We interpret this peak as a small shrinkage of the original fibres upon loss of solvent in the drying process. The xerogel observed in AFM corresponds to this smaller cell dimension, while the crystal studied by *I*–*V* measurements and the gel itself correspond to the cell observed in the single crystal data of **1**.

Rheological assessment using oscillatory sweep tests confirmed **1_MeOH_** to be a gel and not a viscous liquid as the storage modulus value (*G*′) was larger than the loss modulus (*G*′′); the stiffness of the gel was 1.3 kPa (ESI, Fig. S7a†). Frequency-dependent oscillatory measurement showed that *G*′ was dominant across the range of frequencies studied (0.1–100 rad s^–1^) indicating the elastic nature of the gel (ESI, Fig. S7b†). In addition, *G*′ was almost independent of frequency, consistent with the presence of a persistent gel network. The linear viscoelastic region (LVE) of the gel, where *G*′ is independent of the applied strain, was evaluated using an oscillatory strain test and found to be ∼1% of strain (ESI, Fig. S7c†). In this region the structure of the network is not disrupted and remains intact throughout the deformation. The transition from gel to liquid where *G*′ = *G*′′ was observed at 10% strain suggesting that the structure is easily disrupted with deformation, consistent with other supramolecular silver gels.^46^

**Fig. 6:**
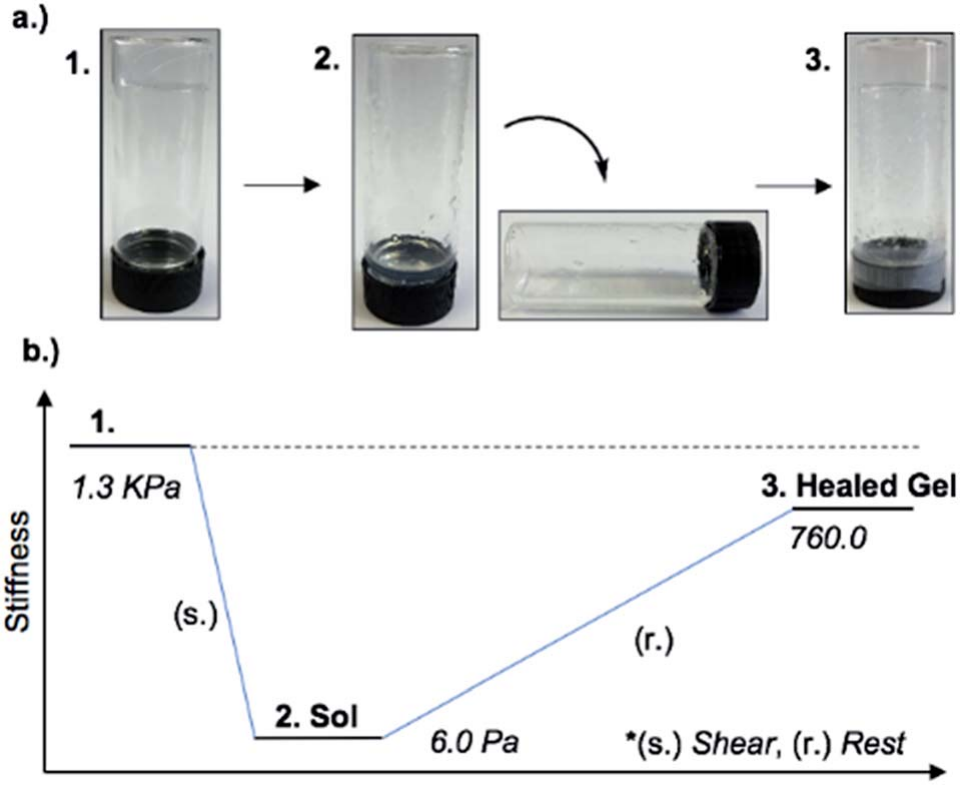
(a) Optical images of the material through application or removal of shear. Inversion test showing the thixotropic nature of **1_MeOH_** ((1) gel, (2) sol and (3) healed gel). (b) A generalised schematic representing the stiffness of the gel through a thixotropic cycle.

An interesting feature of the organogel is its thixotropic nature, *i.e.* the recovery of its gel behaviour after a period of time. **1_MeOH_** collapsed into a sol state with the application of slight shear force (*e.g.* slow vial inversion, minimal vibration) and after approx. 20 minutes reassembled its gel network, as indicated by a simple vial inversion test (Fig. 6). The viscosity of the organogel, studied as a function of shear rate (ESI, Fig. S7d†) showed a shear-thinning behaviour, reducing the viscosity by three orders of magnitude as the shear rate increased; again, typical of a supramolecular gel. In addition, the forward and reverse scans (ESI, Fig. S7d†) did not overlap suggesting that the network is disrupted by the applied shear and cannot recover instantaneously. Furthermore, the gel reduced its stiffness from *G*′ = 1.3 kPa to ∼6 Pa after shear. The recovery of the material was approx. 60% of the initial *G*′ value (760 Pa) after standing for 48 h confirming the self-healing properties and reassembly of the supramolecular network (Fig. 6). Finally, the addition of 1 M urea leads to the rapid collapse of the gel network with the sample converting to the solution state. This and the “self-healing” ability of **1_MeOH_** can be readily understood based on the reversibility of the intermolecular interactions seen in the supramolecular structure of **1**. This behaviour is fully consistent with intermolecular hydrogen bonding being a major contributor to the superstructure of the gelating fibres.

### Electrical conductivity and band structure calculations on a metallo-DNA model

The electrical properties of metallo-DNA have been of interest since reports by Lee *et al.* on M-DNA (M = Co^2+^, Ni^2+^ or Zn^2+^).^37^,^38^ Despite conflicting findings for these systems,^47^–^50^ metallo-DNAs remain putative molecular or nano-wires primarily on account of the linear array of metal ions produced.^22^ For monovalent silver ions the tendency to participate in metallophilic interactions^51^ makes such systems particularly interesting. However, studies on the electrical properties of Ag(i)-DNA are rather few^39^–^42^ and only the most recent work by Galindo *et al.*^36^ is on material that has been crystallographically characterised.

**Fig. 7:**
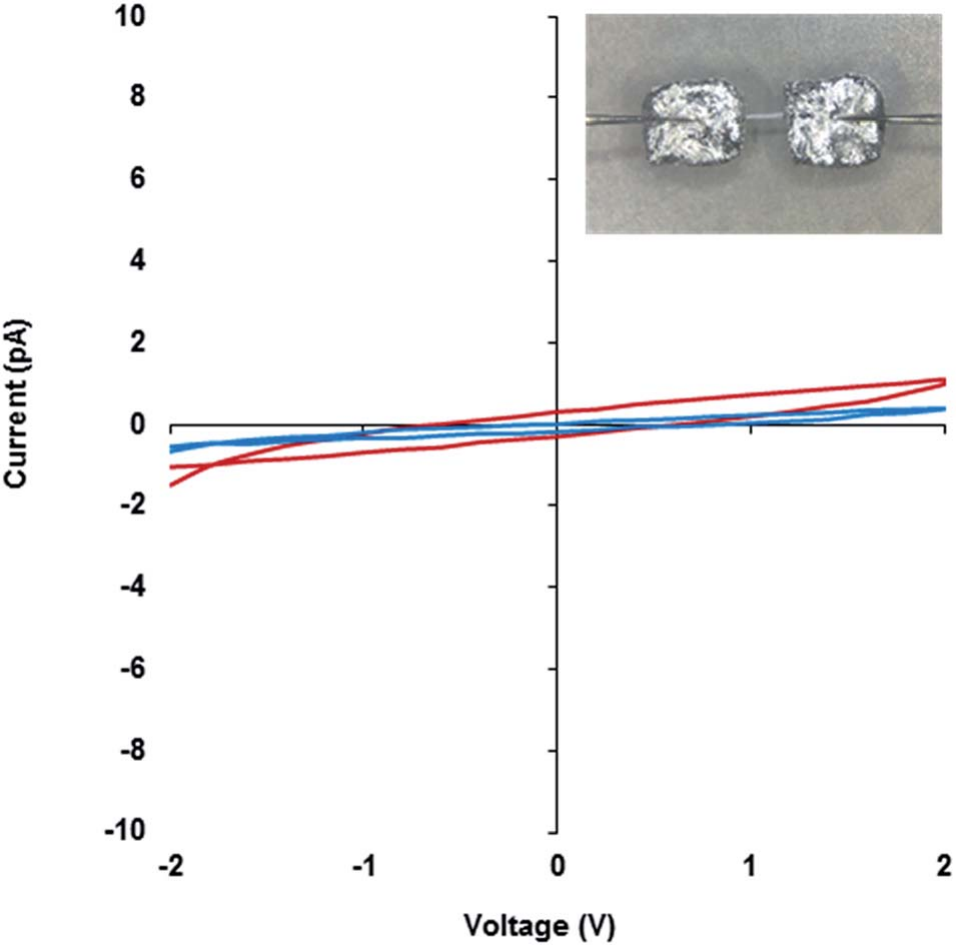
*I*–*V* curves of the **1_MeOH_** xerogel (red) and crystal of **1** (blue). Inset, an optical microscope image of a single crystal of **1** connected to probe station tips through Ga–In eutectic along the crystallographic *c*-axis.

Compound **1** is particularly useful as a structurally well-defined model of a Ag(i)-DNA metallo-array and allows the electrical properties and molecular wire credentials to be addressed. Furthermore, the ability to grow high quality single crystals of **1** allowed us to perform electrical measurements on oriented samples. Single crystals of **1** were mounted onto an electrical probe station and the probe needles contacted the crystal either directly or *via* Ga–In eutectic pads at opposite ends of the long crystallographic *c*-axis. In this orientation any charge passed would flow along the direction of the helical axis (Fig. 7 and ESI, Fig. S13†). *I*–*V* sweeps over a ±2 V range showed only background levels of current (<1 pA) indicating the material is not electrically conducting but a highly effective insulator. Similar results were found for the corresponding xerogel (ESI, Fig. S13†). It is worth restating that the Ag···Ag distances in **1** are within <5% of the metallic distance and rather shorter than those found in Ag(i)-modified oligonucleotides^22^ and similar systems.^35^ Our findings of non-conductivity are in agreement with the conductivity measurements and the electrostatic force microscopy (EFM) experiments of Galindo *et al.* on microcrystals of [Ag(i)(methyl-cytosine)_2_]^+^ that has similarly short Ag···Ag distances.^36^

**Fig. 8:**
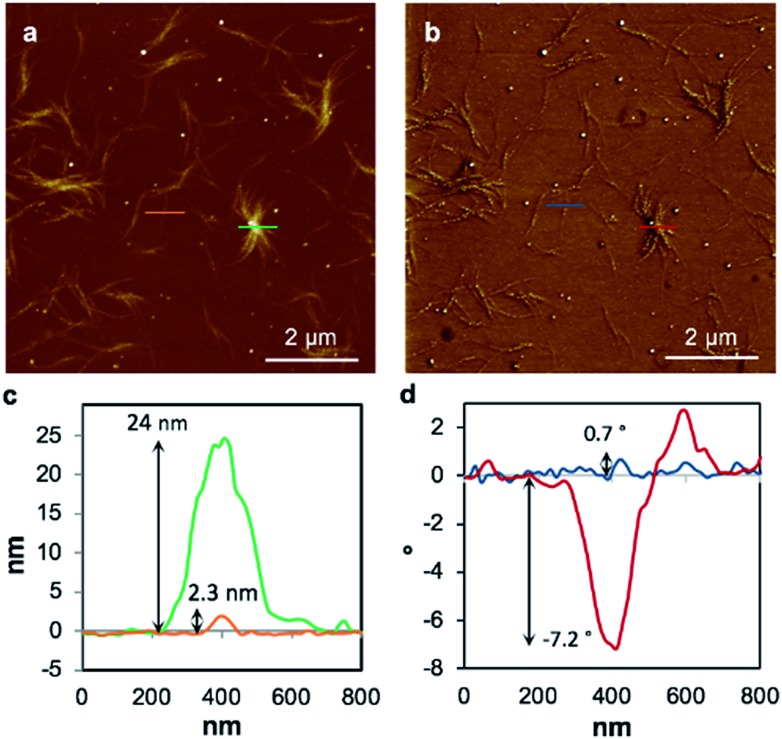
Tapping mode AFM height image of the 20 times diluted **Xe1** (a), the corresponding EFM phase image (b), line profiles corresponding to the green and orange lines in the AFM height image (c) and a line profile corresponding to the blue and red lines in the EFM phase image (d). The EFM image has been captured at applied DC bias +10 V and a lift height of 30 nm.

To examine the charge mobility further, EFM was employed as this can sensitively probe the polarisation of individual fibres. To allow this, the gel **1_MeOH_** was diluted with methanol by a factor of twenty, drop-cast onto a Si was diluted with methanol by a factor of twenty, drop-cast onto a Si〈100〉/200 nm SiO100 was diluted with methanol by a factor of twenty, drop-cast onto a Si〈100〉/200 nm SiO/200 nm SiO_2_ slide, and kept in a methanol-saturated environment to allow self-healing. Then the gel was left to dry in air to form the xerogel, **Xe1**. A control experiment confirms that the fibres in the AFM and EFM images are not derived from solvent impurity (ESI, Fig. S14†). Fig. 8 shows an AFM height image and the corresponding EFM phase image for single fibres as well as aggregated assemblies. An obvious consequence of the dilution is a dramatic thinning and shortening of the fibres (Fig. 8a) indicating that the assembly process is concentration dependent. The individual structures are now typically less than 500 nm long and around 2 nm in height (Fig. 8c), similar to the diameter of an individual duplex. The line profile in Fig. 8d corresponds to the blue line across a single fibre in Fig. 8b and shows a small positive phase shift ∼0.7° (see also ESI, Fig. S15†). This indicates that the fibre is not conductive, again in agreement with the results on single crystals of **1** and the cAFM and EFM experiments of Galindo *et al.*^36^

It is worthwhile to state that STM measurements on single molecule Ag(i)-polydC structures^39^ that show evidence of tunnelling currents are not in contradiction with the absence of conductivity in our measurements on crystals of **1** or fibres of the **Xe1**. The STM measurements demonstrate tunnelling over nm-scale distances, but given the exponential variation of such currents with interelectrode separation, it is quite expected that at the 100 nm scale probed by SCM the conductivity is negligible and even more so for the single crystal *I*–*V* measurements on the mesoscale. In general the linear variation of resistance with electrode separation in a conductive material is a distinct phenomenon from the exponential decay of tunnelling rate in STM or break-junction experiments in which clearly insulating samples may show significant tip currents at short distances.

**Fig. 9:**
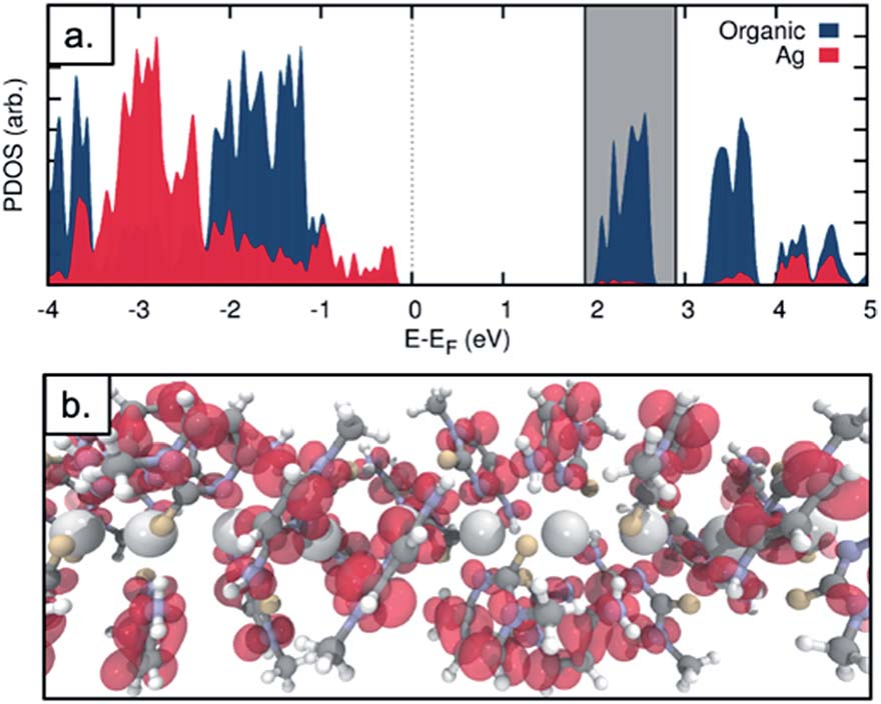
(a) Projected density of states for the Ag chain structure. The density on the central Ag atoms (red) and on the surrounding organic structure (blue) are plotted. (b) Local density of states for an energy window of 1.9–2.9 eV relative to the Fermi energy (shaded grey in figure a).

The observed electrical behaviour is consistent with the calculated local density of states for the ground state system of a single chain of 1* containing five molecules as in the unit cell (where 1* is [Ag(*N*3-(*N*1-methyl-cytosine))_2_]). We plot the ground-state projected density of states on the central silver atoms and the surrounding organic structure in Fig. 9a and find a band-gap at the Fermi energy of 2.502 eV (495.54 nm). Given the well-known shortcomings of the DFT depiction of the electron–electron interactions, causing the underestimation of the band gap,^52^–^55^ we can think of this value as a lower bound and state with a high level of confidence that we do not expect the structure to conduct in its ground state. In addition, this band-gap can help to explain the fact that the solid material is colourless. If a surplus of electrons was introduced – by chemical reduction, for example – the LUMO states of the molecule would be occupied. This would shift the Fermi energy into the LUMO region and allow the conduction electrons to tunnel through the LUMO states. We note that the projected density of states indicates that the majority of the electron density in the LUMO states is found on the organic structure and not the central Ag chain. We plot the Local Density of States (LDOS) for the energy window of 1.9 to 2.9 eV relative to the Fermi level – encompassing the first band of LUMO states (this window is shown as the grey shaded in Fig. 9a). We find that the electron density is almost entirely located on the surrounding organic structure and not the silver chain itself (see Fig. 9b), suggesting that even in the reduced case the structure would not be a metal-based conductor. In fact, however, we find that the material is not stable under these conditions and is transformed into a nanocomposite containing both luminescent Ag clusters and larger particles (*vide infra*).

**Fig. 10:**
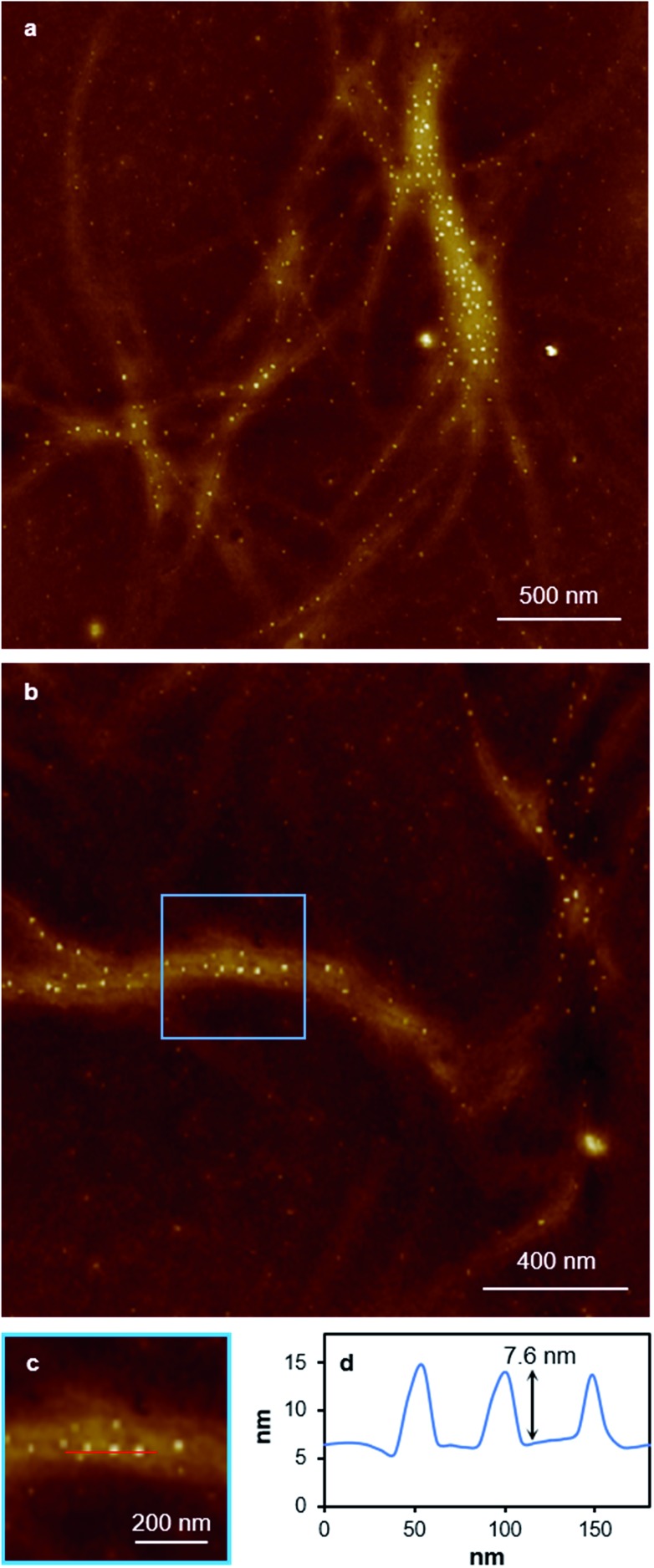
AFM height images of a 20-times diluted sample of xerogel **Xe1**. Large scan range images (a) and zoom-in (b) showing Ag-particles embedded in gel fibres. Zoom-in area (c) showing the location of the line profile, and (d) a line profile corresponding to the red line in the image (c). The line profile along a single fibre in the AFM image (c) shows nanoparticles that protrude from the underlying fibre by ∼7.6 nm with an estimated overall diameter of ∼14 nm.

While the xerogel **Xe1** is not electrically conducting, EFM data indicate that in some parts of the sample, where fibres aggregate, enhanced charge mobility is observed. This can be seen in the EFM phase images as dark regions with corresponding negative phase shift (Fig. 8b and red line Fig. 8d) that varies quadratically with the applied voltage between +10 V and –10 V (ESI, Fig. S15†). It should be noted that negative phase shifts cannot be assigned to merely polarisable structures and the parabolic bias voltage dependence also rules out electrostatic or trapped charge effects which give a linear dependence. Such negative phase shifts are evidence of structures that allow transport of charge away from the immediate vicinity of the tip. AFM also reveals the presence of small particles, likely arising from photoreduction of Ag(i) ions, embedded within fibres (Fig. 10). In parts of the sample these can be quite monodisperse (*ca.* 14 nm total height) and somewhat regularly spaced along individual fibres (Fig. 10b–c). In most cases these metal particles are sufficiently isolated as to not be in contact. They therefore show only positive phase shifts in EFM because they do not provide a pathway for charge to flow away from the tip during the tapping motion. However, in regions where the fibres aggregate, clustering of nanoparticles occurs. This explains the charge-mobile “hot spots” observed by EFM and indicated by the red lines of Fig. 8b and d. These findings are also consistent with the cAFM data on samples of crystalline [Ag(i)(methyl-cytosine)_2_]^+^ reduced using cold H-plasma.^36^

### Photoreduction of a Ag(i)-mediated base pair array and luminescent cluster formation

This observation of nanoparticles prompted us to investigate the reduction behaviour of the material further particularly in light of its relevance to the formation of DNA-templated luminescent silver clusters. Since the first reports by Dickson *et al.*^25^,^26^ these have been increasingly explored as optical materials^28^,^56^ and in bio-sensing applications.^24^ The Ag(i)–cytidine interaction is a key feature in these preparations and homo-cytidine strands have been used to form a range of luminescent Ag cluster species.^27^,^30^,^45^,^57^–^61^ The highly ordered contiguous [cytidine–Ag–cytidine] array such as in **1** and similar compounds^35^,^36^ offer interesting systems for exploring this reactivity and can provide some insight into the nature of the resulting products.

**Fig. 11:**
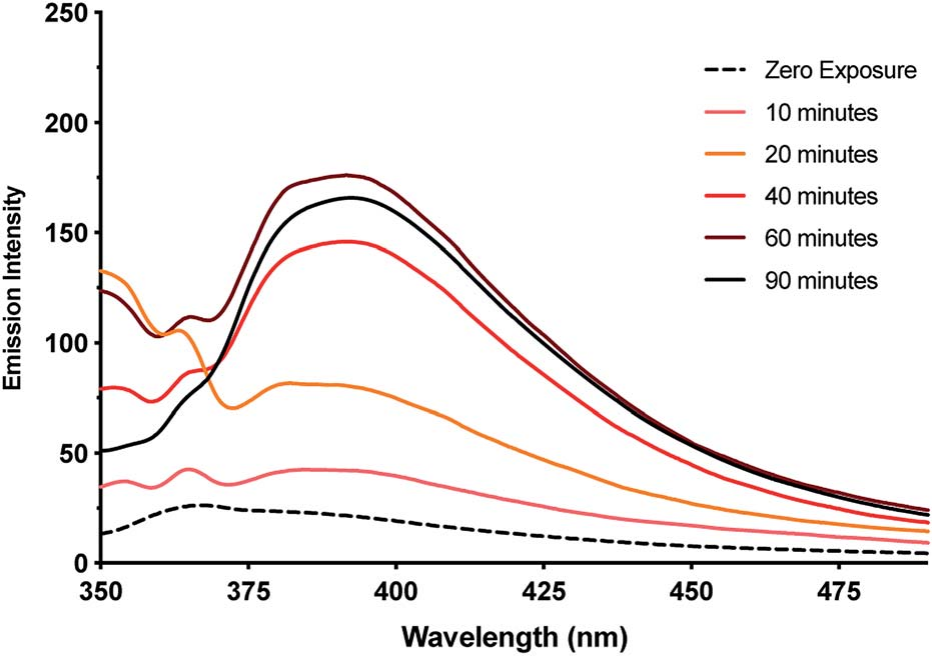
Emission intensity of metallogel **1_MeOH_***versus* exposure time to UV-light (*λ*_Em_: 395 nm, *λ*_Ex_: 330 nm). Inset emission spectra showing the increase in intensity of the emission band up to 60 minutes exposure.

It was found that even freshly-prepared samples of **1_MeOH_** show emission (*λ*_Em_ = 395 nm; *λ*_Ex_ = 330 nm), suggesting that the material is quite photosensitive. Upon UV irradiation (*λ* = 300 nm) samples undergo a colourless-to-red colour change (ESI, Fig. S16†) and the emission intensity increases with exposure times up to 1 h (Fig. 11, ESI, Fig. S17 and S18†). These results are consistent with reduction of the Ag(i) ions in the complex with the formation of quantum-confined silver clusters. The emissive nature indicates that these are sufficiently small, of the order of the Fermi wavelength (∼0.5 nm for Ag, <30 atoms), for a band gap to emerge. Exposure past one hour gave no further increase in the emission intensity and, in fact, a slight diminution was seen. This is consistent with the formation of larger, non-emissive, plasmonic nanoparticles at longer reaction times and the appearance of an absorption band at ∼398 nm supports this (ESI, Fig. S19†).^62^ The emission persisted unchanged for >1 month suggesting the clusters are in a highly stabilising chemical environment.

**Fig. 12:**
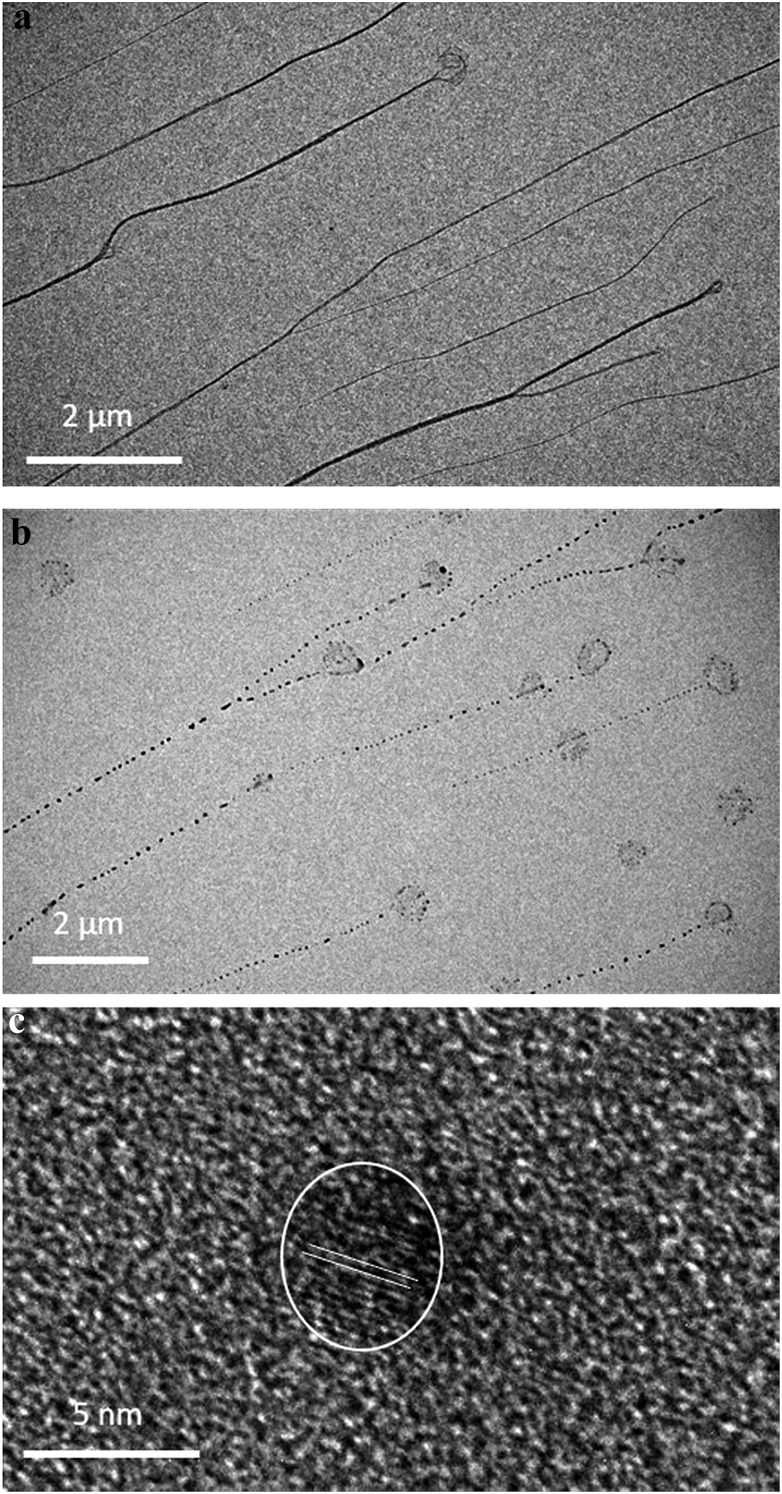
TEM bright field images of gel before and after illumination. (a) Homogeneous fibres in keeping with the regular metallo-array in **1** and (b) after illumination showing the formation of electron-dense nanoparticles along the fibre axis. (c) HR-TEM image of a silver particle (dia. 4.3 nm) with lattice fringe spacing 0.25 nm ± 0.02 corresponding to the interplanar distances of (111).

TEM imaging comparing freshly-prepared and exposed samples illustrates this transformation towards a photoemissive material. For freshly-prepared gel, thinner sections were seen to contain features that can be ascribed to the onset of cluster/particle formation (ESI, Fig. S20†). This is consistent with the AFM studies that show particles embedded in gel fibres (*e.g.*Fig. 10). Particle size analysis of these regions gives a range of 1–4 nm; too large to be emissive but much smaller than the particles observed after exposure. The effect of this is shown in Fig. 12 which highlights the formation of electron-dense particles coincident with the fibre axes giving a heterogeneous appearance. This compares to the homogeneous nature of the fibres prior to exposure as expected for the regular spaced array of metal ions in **1**. High resolution scanning of individual particles shows the expected crystal lattice fringes with typical spacing of 0.25 ± 0.02 nm corresponding to silver metal (Fig. 12 and ESI, Fig. S21†).

## Conclusions

We have shown that the simple metal-mediated base pair [Ag(i)-(*N*3-cytidine)_2_], **1**, formed by coordination of Ag(i) and cytidine, undergoes a remarkable self-assembly process to form a supramolecular double helix. This process is driven by a combination of complementary hydrogen bonding, hydrophobic and metallophilic interactions. The reversibility of these interactions allows the material to self-repair when in gel-form. The results highlight the tendency of these building blocks to intrinsically adopt structures similar to the corresponding biopolymer with the assembly being closely analogous to that of metallo-DNAs.^22^ This comparability allows insight into the reactivity and properties of this class of material, not least the question of electrical conductivity. Though **1** has some of the shortest argentophilic interactions reported, close in fact to the metallic radii of 2.88 Å, the material is electrically insulating due to the filled valence band and sizable band gap. While, in principle, chemical reduction could enhance the conductivity of **1**, instead the compound readily deposits silver, as expected. In fact, the assembled “poly-cytidine” array is a highly effective precursor for the formation of luminescent clusters supporting proposals of the importance of cytidine in the synthesis of DNA-stabilised silver clusters. We would suggest that observed regions of “high” charge mobility in such samples are, in fact, due to the clustering of silver particles formed from reduction of the Ag(i) ions in the complex. Finally, while the complementarity between silver chemistry and nucleic acids, and their components, offers great potential for encoding new functions and reactivity into DNA-based materials, a deeper understanding of the interactions and stabilisation of zero-valent and mixed valent silver species will be important.^23^,^30^

## Abbreviations

LVE: Linear viscoelastic region
PDOS: Projected density of states

## Conflicts of interest

There are no conflicts to declare.

## Funding sources

Newcastle University (funded studentship to LM).

## Supplementary Material

Supplementary information

Crystal structure data
