# Supplementary material for: Addressing the properties of “Metallo-DNA” with a Ag(i)-mediated supramolecular duplex
Source: Chem Sci. 2019 Feb 8;10(11):3186–95. doi: 10.1039/c8sc05103h (PMC6429620; doi:10.1039/c8sc05103h)
Supplement: Supplementary file 1 [file SC-010-C8SC05103H-s001.pdf]

Electronic Supplementary Material (ESI) for Chemical Science.
This journal is © The Royal Society of Chemistry 2019

Addressing the properties of “metallo-DNA” with a Ag(I)-mediated supramolecular duplex

Liam Mistry,[a] Osama El-Zubir,[a] Gema Dura,[a] William Clegg,[a] Paul G. Waddell,[a] Thomas Pope,[a] Werner A. Hofer,[a] Nick G. Wright,[b] Benjamin R. Horrocks,[a] Andrew Houlton[a]*

[a] Chemical Nanoscience Laboratory, Chemistry, School of Natural and Environmental Sciences, Newcastle University, Newcastle upon Tyne, UK, NE1 7RU
[b] School of Engineering, Newcastle University, Newcastle upon Tyne, NE1 7RU

Supporting Information

Table of Content

GENERAL METHODOLOGY 1
EXPERIMENTAL 1; SYNTHESIS AND CRYSTALLOGRAPHIC DATA. 2
EXPERIMENTAL 1_{MEOH}; SYNTHESIS AND SPECTROSCOPIC DATA. 6
RHEOLOGY 9
MICROSCOPY, INCLUDING SEM AND AFM IMAGES. 10
CONDUCTIVITY MEASUREMENTS, IV AND EFM. 13
REDUCTION BEHAVIOR. 18
BAND STRUCTURE CALCULATIONS 21

General Methodology

Reagents were used as obtained from Sigma-Aldrich unless otherwise stated.

NANOpure® deionised water (18 MΩ cm resistivity) was obtained from a NANOpure® DIamondTM Life Science ultrapure water system equipped with a DIamondTM RO Reverse Osmosis System (Barnstead International).

NMR spectra were recorded using Bruker 400 MHz spectrometer at RT. ^{1}H-NMR shifts were referenced to D_2O [^{1}H-NMR, δ (D_2O) = 4.80 ppm].

Experimental IR spectra in the solid state were measured with a Shimadzu IRAffinity-1S spectrometer using LabSolutions RF software. Spectra were recorded over the 400 – 4000 cm^{-1} range with 50 scans.

UV-Vis measurements (in the range of 190 - 850 nm) were conducted using a Thermo Scientific™ NanoDrop™ One^{C} Microvolume spectrophotometer. A 1.5 μl sample droplet was prepared with an auto-ranging pathlength 0.03 – 1.00 mm (wavelength accuracy ± 1 nm).

Microanalyses were carried out by the Elemental Analysis Service in the School of Human Sciences, London Metropolitan University. Solutions were freeze dried to remove solvent prior to analysis. The individual C, H, N values did not differ by more than ±0.3 %. The mean C, H, N values are reported.

Emission spectroscopy (in the range of 300 – 800 nm) in a gel or liquid state were measured using a Shimadzu RF-6000 Spectro Fluorophotometer using a quartz cuvette (50 µl) with a path length of 1.5 mm. Scan intervals were acquired every 0.5 nm at a scan speed of 600 nm/min, within an emission and excitation bandwidth of 5.0 nm. The system was controlled using LabSolutions RF software.

Circular dichroism spectra were measured in a high precision quartz SUPRASIL (Hellma Analytics®) cell with a light path of 0.1 mm. Data was acquired using a JAS.CO J-810 spectropolarimeter under nitrogen (5-10 ml/min) connected to a PTC-423S temperature controller maintaining 25°C using a Julabo water bath.

For high-resolution electrospray ionisation mass spectrometry (ESI-MS) studies, xerogel samples were mixed in ultrapure (type 1) water at concentrations in the order of 100 ng ml^{-1}. Measurements were made on a Waters Micromass LCT Premier TOF Mass Spectrometer and data was processed using the Mmass software (version 5.5.0).

Experimental 1; Synthesis and Crystallographic Data.

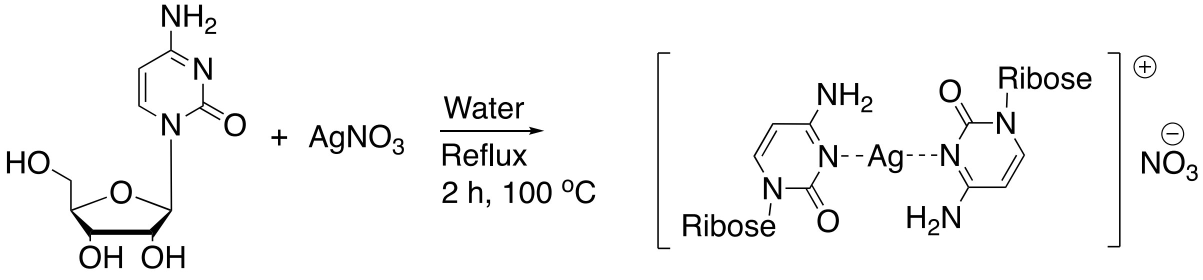

(2 equiv.) (1 equiv.)

Synthesis of [Ag(*N3*-cytidine)$_2$][NO_3], (**1**)

Cytidine (200.0 mg, 0.88 mmol, 2.0 equiv.) was dissolved in water (4.5 ml). To this solution, another aqueous solution (0.5 ml) of $AgNO_3$ (74.7 mg, 0.44 mmol, 1.0 equiv.) was added slowly. The mixture was heated under reflux (100 °C) for 2 h in the dark. The solution was filtered while still warm; Long needle-like crystals were grown over a period of 14-days at room temperature using a thermal vapor diffusion chamber with acetonitrile as anti-solvent. The yield of crystals was 67%. ^{1}H NMR (400 MHz, Deuterium Oxide) δ 7.91 (d, *J* = 7.6 Hz, 1H), 6.09 (d, *J* = 7.5 Hz, 1H), 5.89 (d, *J* = 3.8 Hz, 1H), 4.33 – 4.25 (m, 1H), 4.19 (t, *J* = 5.7 Hz, 1H), 4.16 – 4.10 (m, 1H), 3.95 (dd, *J* = 12.7 Hz, 1H), 3.81 (dd, *J* = 12.8, 4.2 Hz, 1H); IR: ṽ = 3337, 3208, 2945, 1643, 1606, 1529, 1504, 1330, 1280, 1209, 1101, 1043, 950, 903, 870, 772, 717, 600 and 407 cm^{-1}; ESI-MS ($C_{18}H_{26}AgN_6O_{10}$) calculated: 593.0800, Experimental: 593.0957 (m/z); Elemental analysis ([Ag(*N3*-cytidine)$_2$]·NO_3·$2H_20$) Calculated: C - 31.23%, H - 4.37%, N - 14.16%, Experimental: C – 31.79%, H - 4.25%, N - 13.91%.

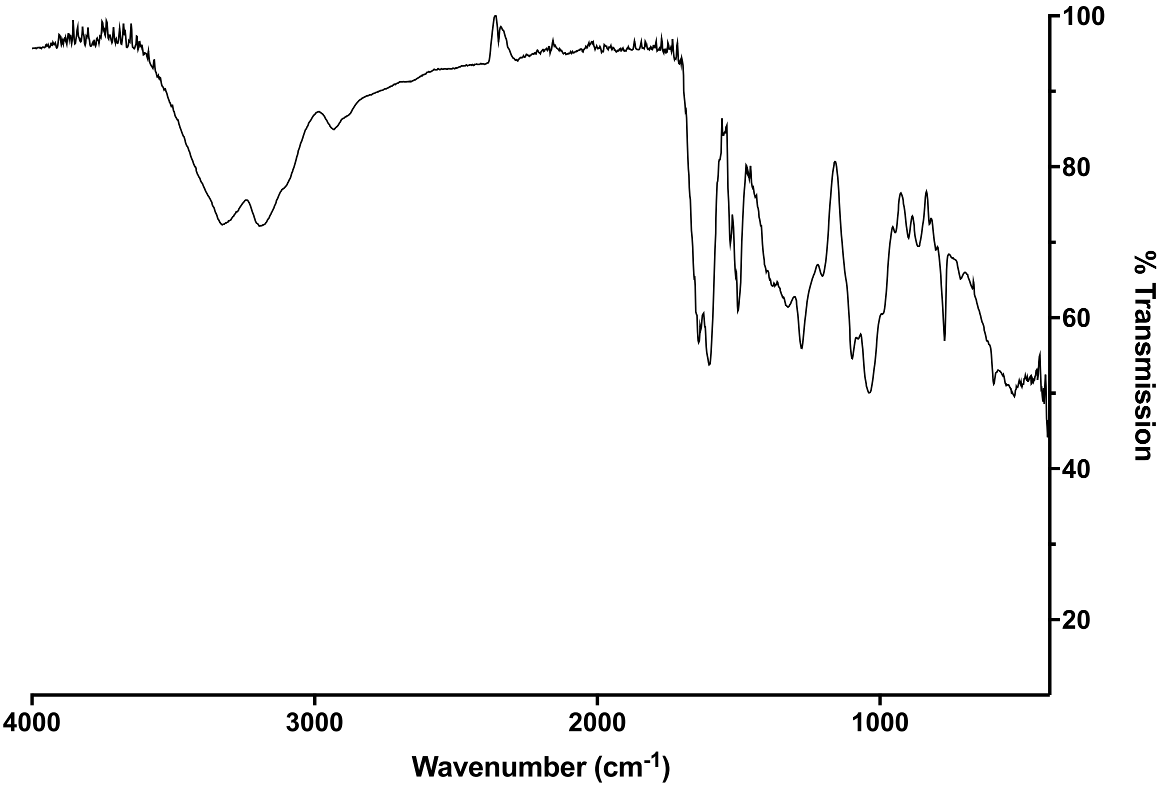

Figure S1. FTIR data of complex **1**, over the 400 – 4000 cm^{-1} range.

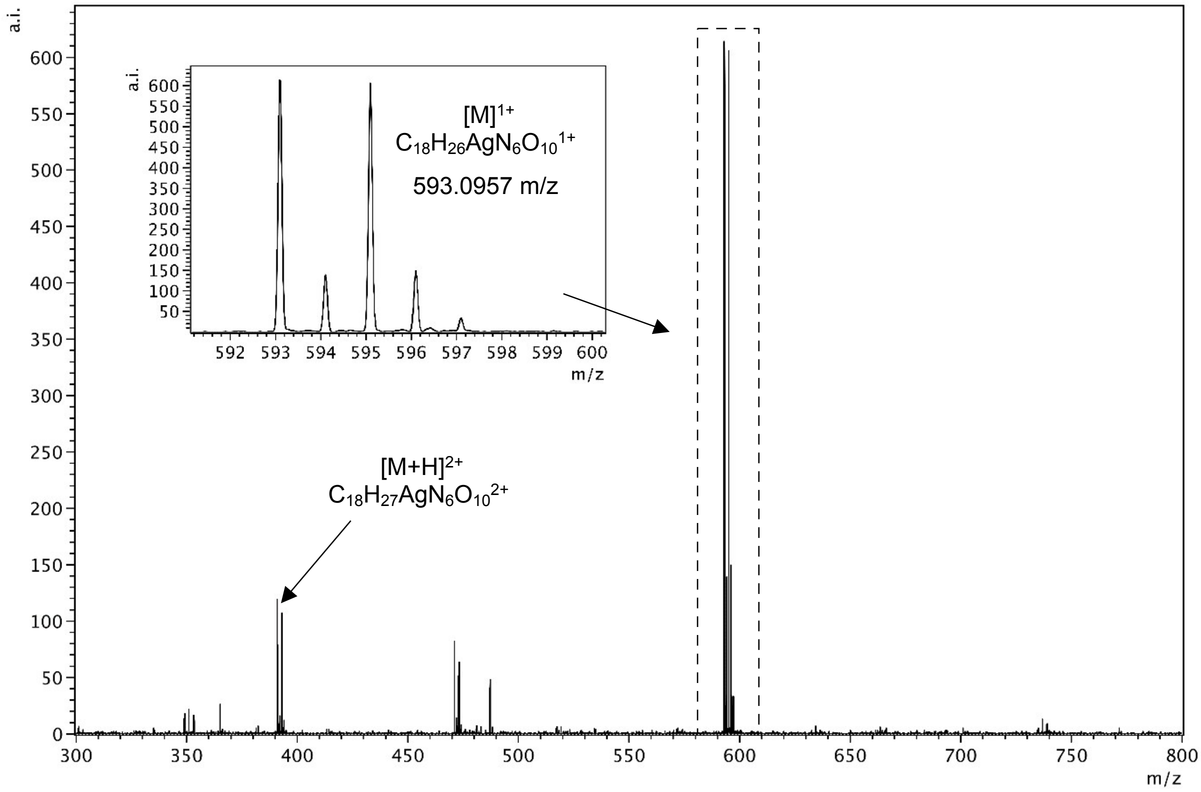

Figure S2. ESI-MS data measured for **1** collected in high resolution positive-ion mode.

X-Ray Crystal Structure Determination for complex **1.**

The crystallographic study of **1** was hampered by a number of factors. Available crystals were too small and weakly diffracting for investigation using standard in-house facilities, even with enhanced microsource X-ray intensities. The weakness of the diffraction was subsequently found to be exacerbated by extensive structural disorder and pseudo-symmetry. After repeated attempts an adequate set of single-crystal diffraction data was obtained at beamline I19 of Diamond Light Source,[1] providing a reliable unit cell geometry and a clear indication of the space group (one of an enantiomeric pair), which had been uncertain from earlier attempts, possibly because of some merohedral twinning.

Standard multiscan corrections were applied for absorption and for incident beam intensity variations.[2] The positions of Ag atoms were readily found by a variety of routine direct and heavy-atom methods. These, however, are arranged in a regular and almost linear chain along the crystallographic *c* axis, with 5 Ag atoms in the asymmetric unit. This leads to a dominance of the diffraction pattern by strong reflections with $l = 5n$. Use of all available routine methods for structure solution failed due to the pseudo-symmetry of the helical structure of the polymeric cation chain, which was subsequently found to display approximate non-crystallographic fivefold screw axis symmetry; the threefold crystallographic screw axis relate cation chains to each other. The structure was eventually solved with successive manual selection of possible ligand atom positions from the multiple images in electron density maps insufficiently phased by the silver atoms, relying on the recognition of fragments of cytidine rings.

To aid refinement, geometrical similarity restraints were applied to the bond lengths and angles of the 10 ligands in the asymmetric unit, together with similarity and rigid-bond restraints on their anisotropic displacement parameters.[3] H atoms were included in geometrically calculated positions and refined with a riding model; those on OH groups were allocated to give a reasonably self-consistent set of positions appropriate for hydrogen bonds where these could be identified. Reliable positions could not be found for the nitrate counter-anions or solvent molecules, which occupy large apparent voids between cation polymer chains; these were treated by the SQUEEZE procedure of PLATON.[4] Nitrate anions, but not solvent molecules, are included in the chemical formula and calculated physical properties.

Resonant ('anomalous') scattering effects of the silver atoms are significant and provide confirmation of the absolute configuration of the overall structure, consistent with that known for the ligand sugar component,[5] giving a refined value of 0.08(4) for the absolute structure parameter.

$C_{18}H_{26}AgN_6O_{10}^+$ NO_3^-, $M = 656.3$, trigonal, space group $P3_2$, $a = b = 30.382(7)$, $c = 14.976(3)$ Å, $V = 11972(6)$ Å^3, $Z = 15$ ($Z' = 5$), crystal size $0.194 \times 0.016 \times 0.014$ mm^3, $T = 100$ K, λ(synchrotron) = 0.6889 Å, 93293 reflections measured, 26785 unique ($R_{int} = 0.0718$), transmission 0.86–0.99; R (F, $F^2 > 2\sigma$) = 0.0904, R_w (F^2, all data) = 0.2777, goodness-of-fit on $F^2 = 0.955$, for 1576 refined parameters and 4509 restraints, final difference map peaks and holes between 1.53 and -0.66 e Å^{-3}.

Table S1. Crystal data and structure refinement parameters for [Ag$^{(I)}$-*N3*-(Cytidine)$_2$]·MeCN·H_2O

Identification code	aho170073
Chemical formula (moiety)	$C_{18}H_{26}AgN_6O_{10}^+ \cdot NO_3$
Chemical formula (total)	$C_{18}H_{26}AgN_7O_{13}$
Formula weight	656.33
Temperature	100(2) K

Radiation, wavelength	synchrotron, 0.6889 Å	
Crystal system, space group	trigonal, $P3_2$	
Unit cell parameters	a = 30.382(7) Å	α = 90°
	b = 30.382(7) Å	β = 90°
	c = 14.976(3) Å	γ = 120°
Cell volume	11972(6) $Å^3$	
Z	15	
Calculated density	1.366 g/cm^3	
Absorption coefficient μ	0.644 mm^1	
F(000)	5010	
Crystal color and size	colorless, 0.194, 0.016, 0.014 mm^3	
Reflections for cell refinement	9382 (θ range 2.2 to 23.4°)	
Data collection method	fixed-χ diffractometer with Pilatus 2M detector<br>narrow-frame ω and ϕ scans	
θ range for data collection	0.8 to 24.1°	
Index ranges	h 35 to 34, k 35 to 35, l 17 to 17	
Completeness to θ = 24.1°	98.7 %	
Reflections collected	93293	
Independent reflections	26785 (R_{int} = 0.0718)	
Reflections with F^2>2σ	14630	
Absorption correction	multi-scan	
Min. and max. transmission	0.860 and 0.990	
Structure solution	Dual-space interative method	
Refinement method	Full-matrix least-squares on F^2	
Weighting parameters a, b	0.1772, 0	
Data / restraints / parameters	26785 / 4509 / 1576	
Final R indices [F^2>2σ]	R1 = 0.0904, wR2 = 0.2500	
R indices (all data)	R1 = 0.1248, wR2 = 0.2777	
Goodness-of-fit on F^2	0.955	
Absolute structure parameter	0.08(4)	
Largest and mean shift/su	0.017 and 0.000	
Largest diff. peak and hole	+1.53 and -0.66 e $Å^3$	

References

1. (a) Allan, D. R.; Nowell, H.; Barnett, S. A.; Warren, M. R.; Wilcox, A.; Christensen, J.; Saunders, L. K.; Peach, A.; Hooper, M. T.; Zaja, L.; Patel, S.; Cahill, L.; Marshall, R.; Trimnell, S.; Foster, A. J.; Bates, T.; Lay, S.; Williams, M. A.; Hathaway, P. V.; Winter, G.; Gerstel, M.; Wooley, R. W. *Crystals* 2017, ***7***, 336. (b) Johnson, N. T.; Waddell, P. G.; Clegg, W.; Probert, M. R. *Crystals* 2017, ***7***, 360.
2. Krause, L.; Herbst-Irmer, R.; Sheldrick, G. M.; Stalke, D. *J. Appl. Crystallogr.* 2015, ***48***, 3.
3. Sheldrick, G. M. *Acta Crystallogr., Sect. C* 2015, ***71***, 3.
4. Spek, A. L. *Acta Crystallogr., Sect. C* 2015, ***71***, 9.
5. Flack, H. D. *Acta Crystallogr., Sect. A* 1983, ***39***, 876. (b) Parsons, S.; Flack, H. D.; Wagner, T. *Acta Crystallogr., Sect. B* 2013, ***69***, 249.

Experimental 1_{MeOH}; Synthesis and Spectroscopic Data.

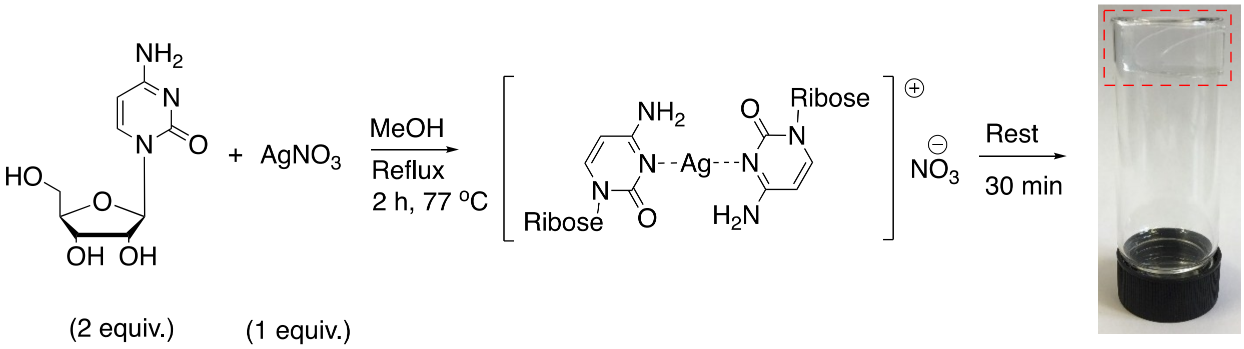

Synthesis of [Ag(*N3*-cytidine)$_2$][NO_3], (**1**$_{MeOH}$) gel.

Cytidine (200.0 mg, 0.88 mmol, 2.0 equiv.) was dissolved in methanol (25 ml). To this solution, another solution of $AgNO_3$ (74.7 mg, 0.44 mmol, 1.0 equiv.) in methanol (25 ml) was added slowly. The mixture was heated under reflux (77 °C) for 2 h in the dark. The solution was filtered while still warm, then left to stand at room-temperature where a sample spanning colourless gel formed over 30 minutes. Conversion of **1**$_{MeOH}$ to the corresponding xerogel (**Xe1**) was achieved by either rapidly cooling the gel with liquid nitrogen, followed by freeze-drying using a Thermo Electron Corporation Heto PowerDry LL3000 Freeze Dryer or by allowing solvent evaporation in air. IR: $\tilde{\nu}$ = 3340, 3201, 2943, 1643, 1604, 1528, 1504, 1338, 1280, 1207, 1099, 1049, 995, 948, 902, 867, 771, 717, 597 and 409 cm^{-1}; ESI-MS ($C_{18}H_{26}AgN_6O_{10}$) calculated: 593.0800, Experimental: 593.0712 (m/z); Elemental analysis ([Ag(*N3*-cytidine)$_2$]·NO_3·$2H_2O$) Calculated: C - 31.23%, H - 4.37%, N - 14.16%, Experimental: C - 31.78%, H - 4.33%, N - 13.84%.

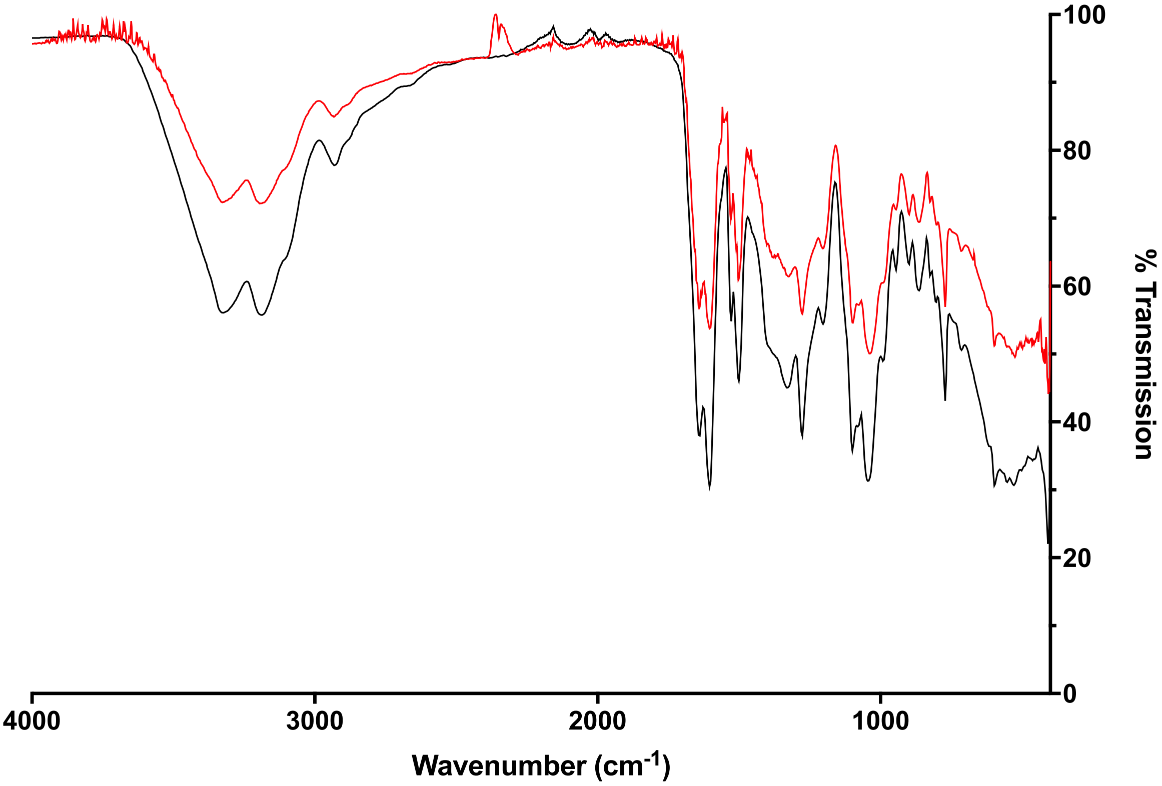

Figure S3. FTIR data of the solid **1** (red line) *vs.* xerogel **Xe1** (black line)

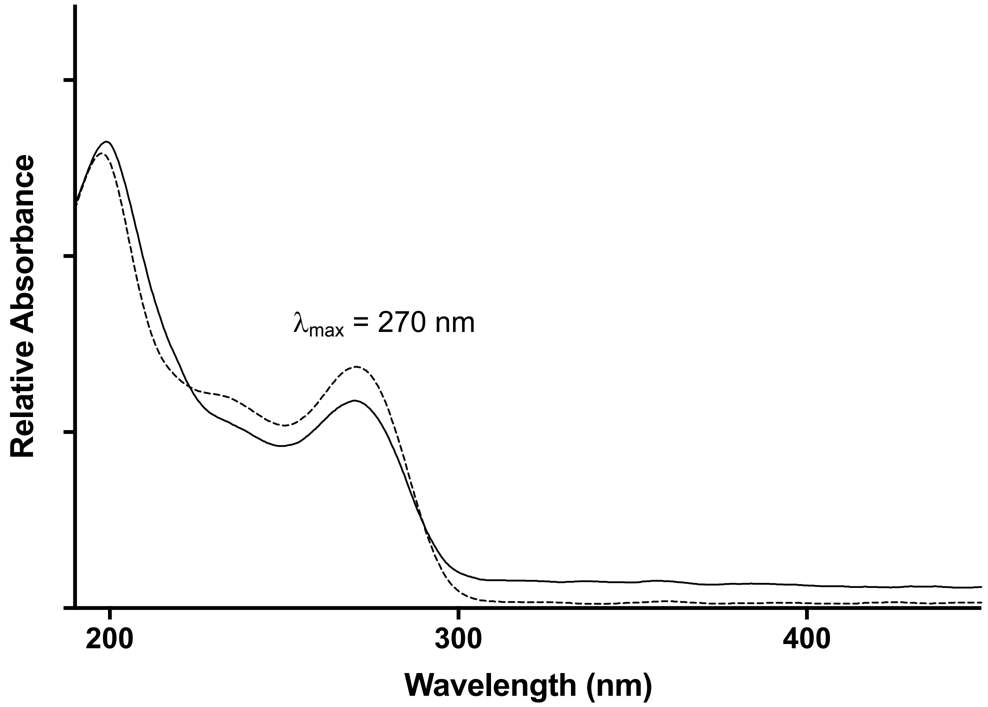

Figure S4. UV-vis spectra of $\mathbf{1}_{MeOH}$ (solid line) *vs.* cytidine (dotted line).

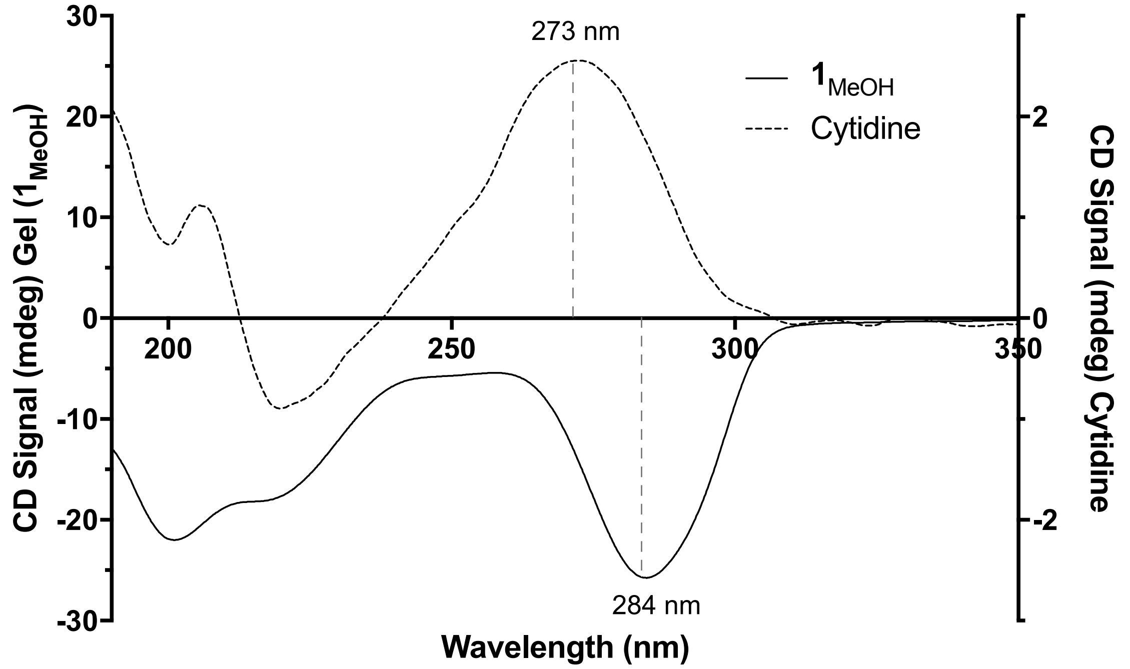

Figure S5. Circular Dichroism spectra for cytidine (dotted line) and $\mathbf{1}_{MeOH}$ (solid line) at concentrations of 4 mg/ml.

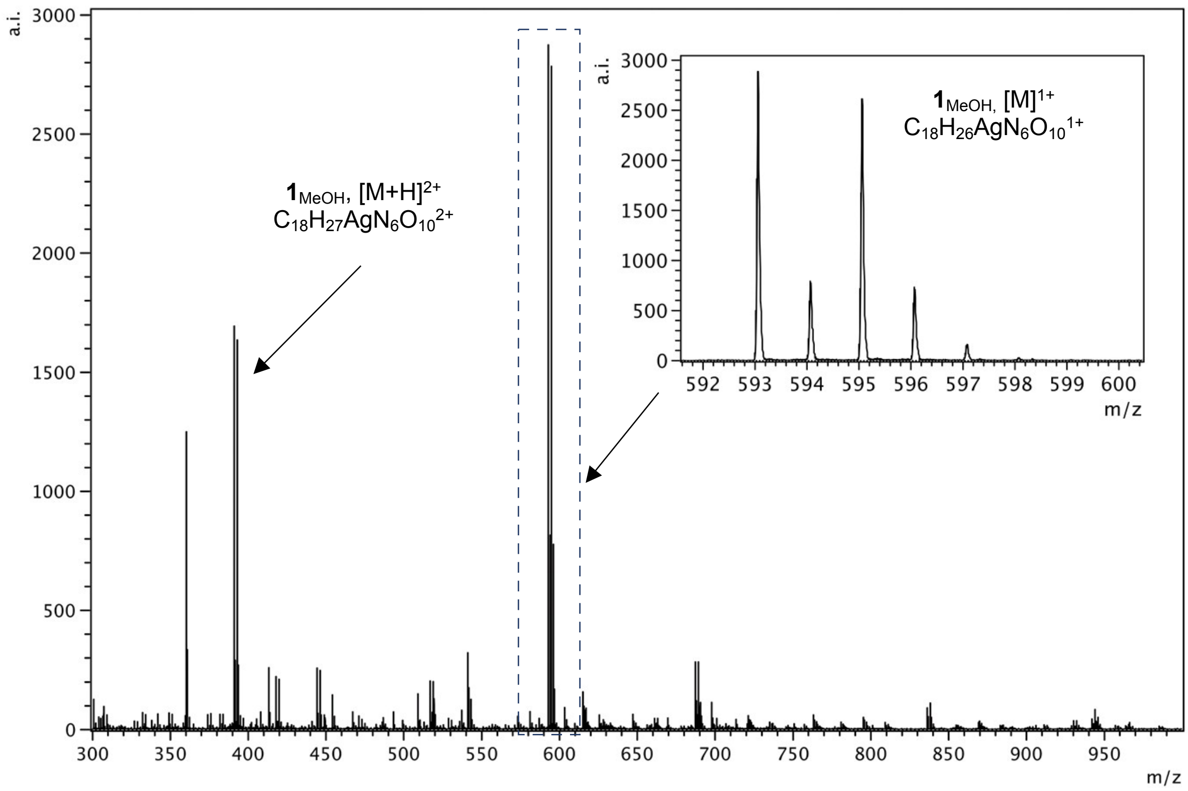

Figure S6. ESI-MS data measured for $\mathbf{1}_{MeOH}$, collected in high resolution positive-ion mode.

Rheology

Rheological measurements were performed with a HR-2 Discovery Hybrid Rheometer (TA Instruments) with a standard steel parallel-plate geometry of 20 mm diameter with a gap of 1 mm. The strain and the frequency were set to 1% and 1 Hz, respectively.

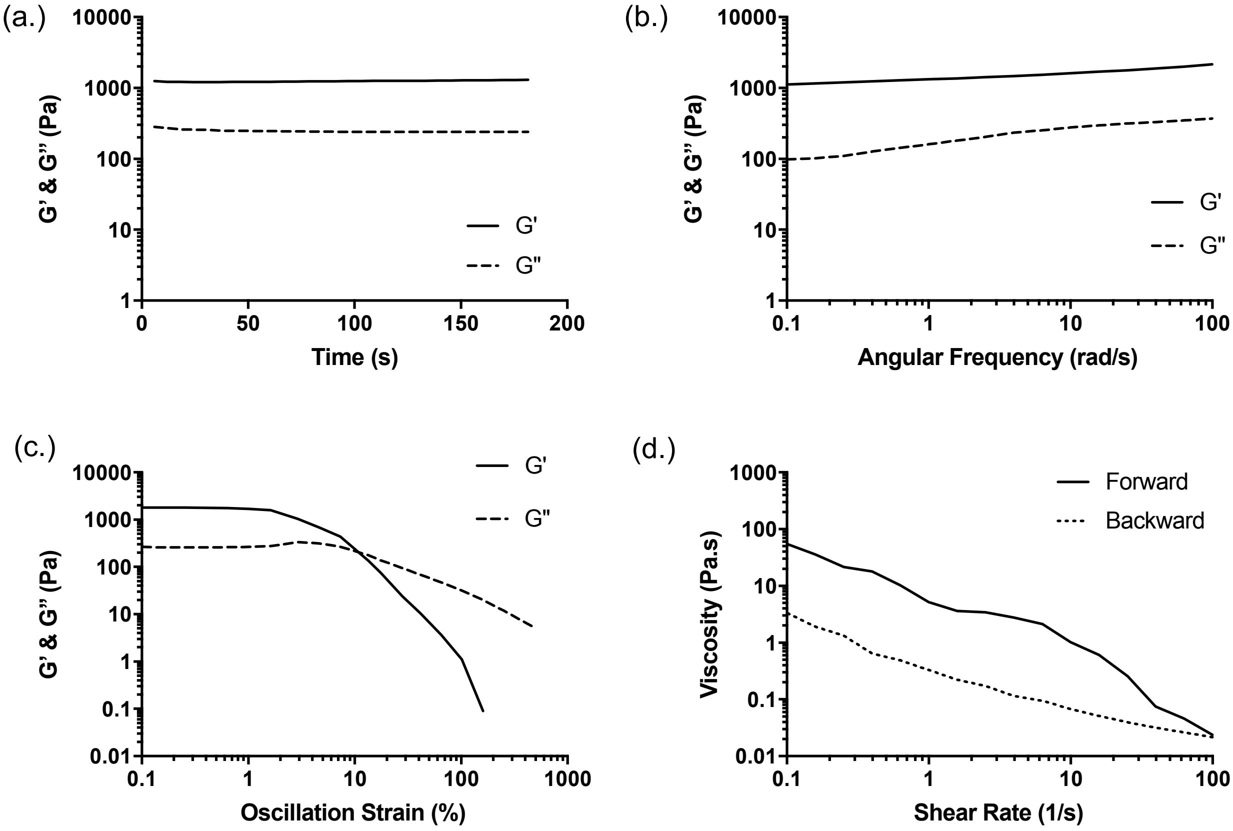

Figure S7. (a.) Rheology time sweep of $\mathbf{1}_{MeOH}$ gel at 4.0 mg mL^{-1} performed at 1% strain, 1 Hz; (b.) Frequency sweep of 4.0 mg mL^{-1} metallogel ($\mathbf{1}_{MeOH}$) conducted at 1% strain from 0.1 to 100 rad/s; (c.) Strain sweep of metallogel ($\mathbf{1}_{MeOH}$) at 4.0 mg mL^{-1} conducted at 1 Hz, from 0.1 to 500 % of strain. G' = G'' point, transition sol-gel; (d.) Viscosity versus shear rate profile between 0.1 and 100 s^{-1}

Microscopy, including SEM and AFM images.

SEM samples of xerogel **Xe1** were mounted on an aluminum stub with Achesons Silver Dag and then dried overnight. Examined using a TESCAN VEGA LMU Scanning Electron Microscope, housed within EM Research Services, Newcastle University. Digital images collected with TESCAN supplied software.

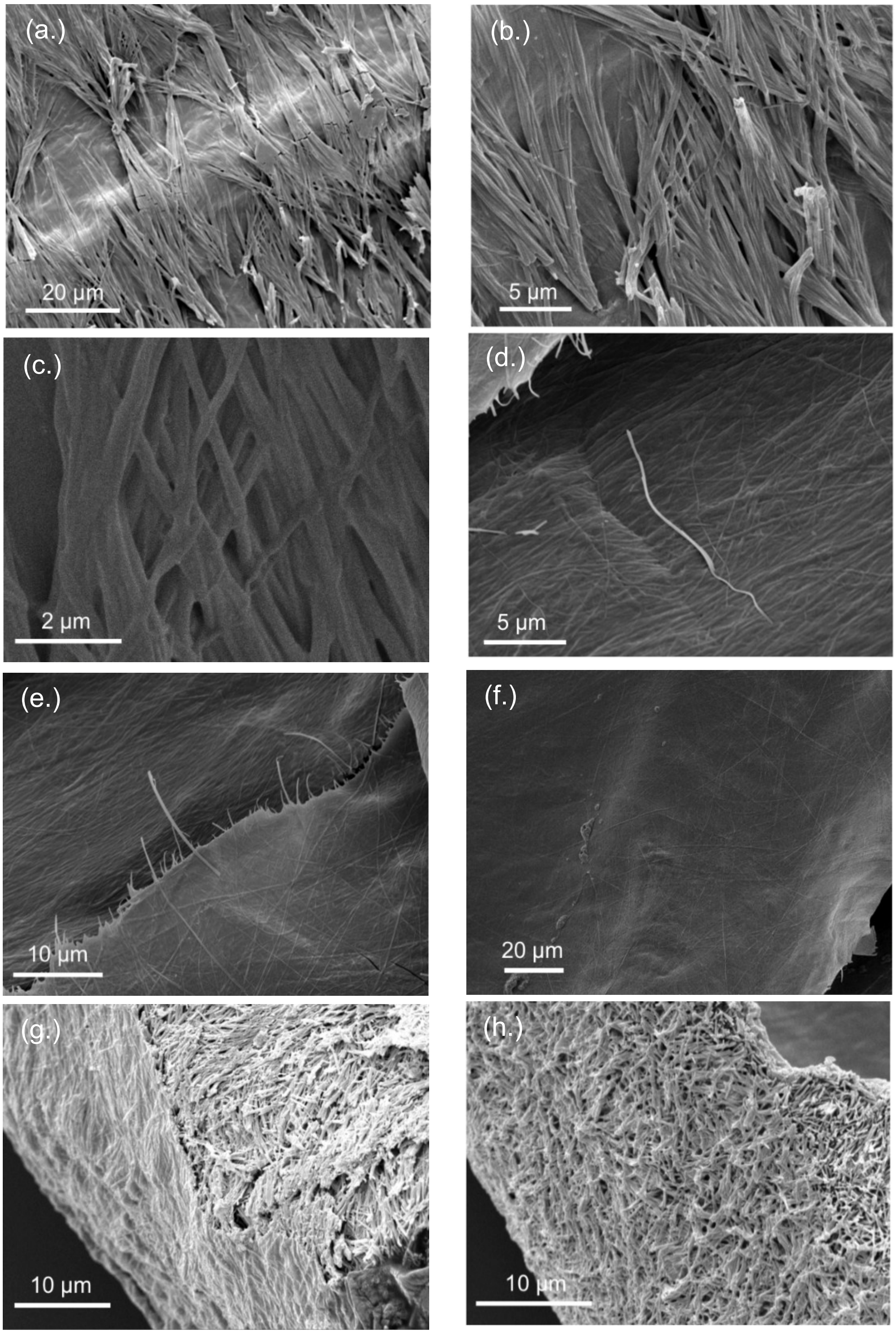

Figure S8. SEM images of xerogel **Xe1** showing long fibres > 6 μm in length; (a. - f.) top-down view of the surface of the xerogel; (g. – h.) Side-on view showing the voids within the network.

Atomic force microscopy (AFM). A Multimode 8 atomic force microscope with Nanoscope V controller and a "E" scanner (Bruker, Germany) was used for acquiring AFM height images. Nanoscope software version 9.1 was used to control the microscope. The system was operated in a peak force tapping mode in air (ScanAsyst) at ultra-low forces to minimise damage to the **Xe1** fibres. An isolation table/acoustic enclosure (Veeco Inc., Metrology Group) was used to decrease vibrational noise. Silicon tips on V-shaped silicon nitride cantilevers (ScanAsyst-Air, Bruker) were used for imaging. The nominal tip radius was approximately 2 nm, resonant frequency 70 kHz, and spring constant k ~0.4 N/m. The AFM height images were analysed with NanoScope Analysis 1.5 software (Bruker). The xerogel sample **Xe1** was prepared by drop-casting 1 μl of $\mathbf{1}_{MeOH}$ gel onto a clean silicon wafer. The sample was kept in a 9 ml closed glass vial for 3 days to allow the self-healing of the gel. Gel transferred to the silicon surface by micropipette led to loss of the fibrous structure in keeping with the thixotropic nature of the gel. If the $\mathbf{1}_{MeOH}$ complex was dried quickly on a silicon surface immediately after it was transferred, it appears in the form of a film of dried material in the AFM images.

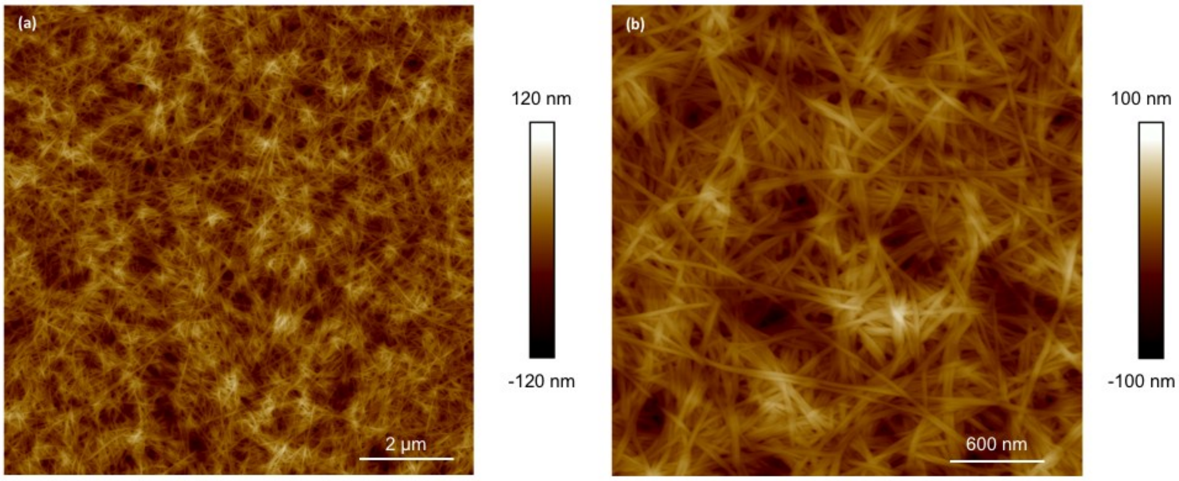

Figure S9. AFM images of **Xe1** drop-cast onto a silicon wafer. Large-scan (a) and a zoom area (b) of AFM height images of **Xe1**.

AFM Height Analysis – The mean diameter of the fibres was estimated using AFM images of the **Xe1**. First, the distribution of heights in the image was determined using Gwyddion[1] with a bin width of 0.188 nm; it is important the bin width is much smaller than the typical feature size. This distribution is shown in Figure 12(a). Next, a pseudo-Voigt function was fitted to the measured distribution by a standard least-squares method and the residual was plotted in Figure 12(b). Clear peaks (labelled 1-6) are seen in the residual which are a result of unresolved components in Figure 12(a) originating from the stacking of fibres in the AFM image. The mean fibre diameter can then be obtained by linear regression of the heights at the peaks of Figure 12(b) against an integer index. This is shown in Figure 12(c) and the fibre diameter was estimated to be 6.3 +/- 0.45 nm.

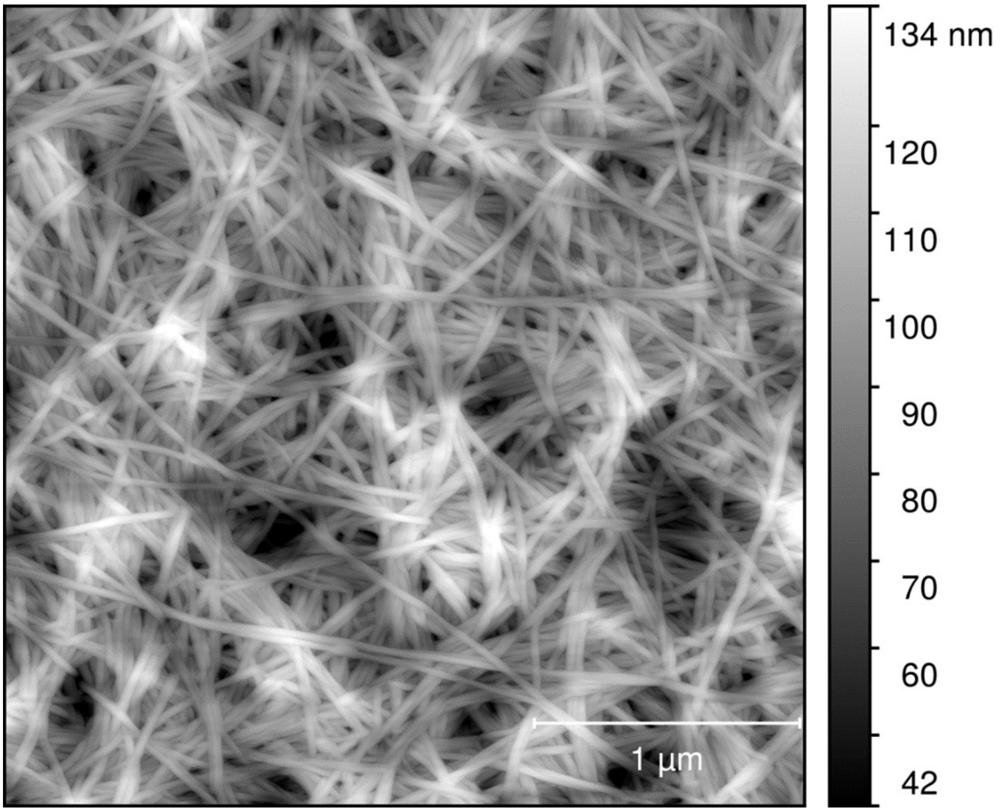

Figure S10. AFM images of **Xe1** drop-cast onto a silicon wafer adjusted to greyscale for analysis.

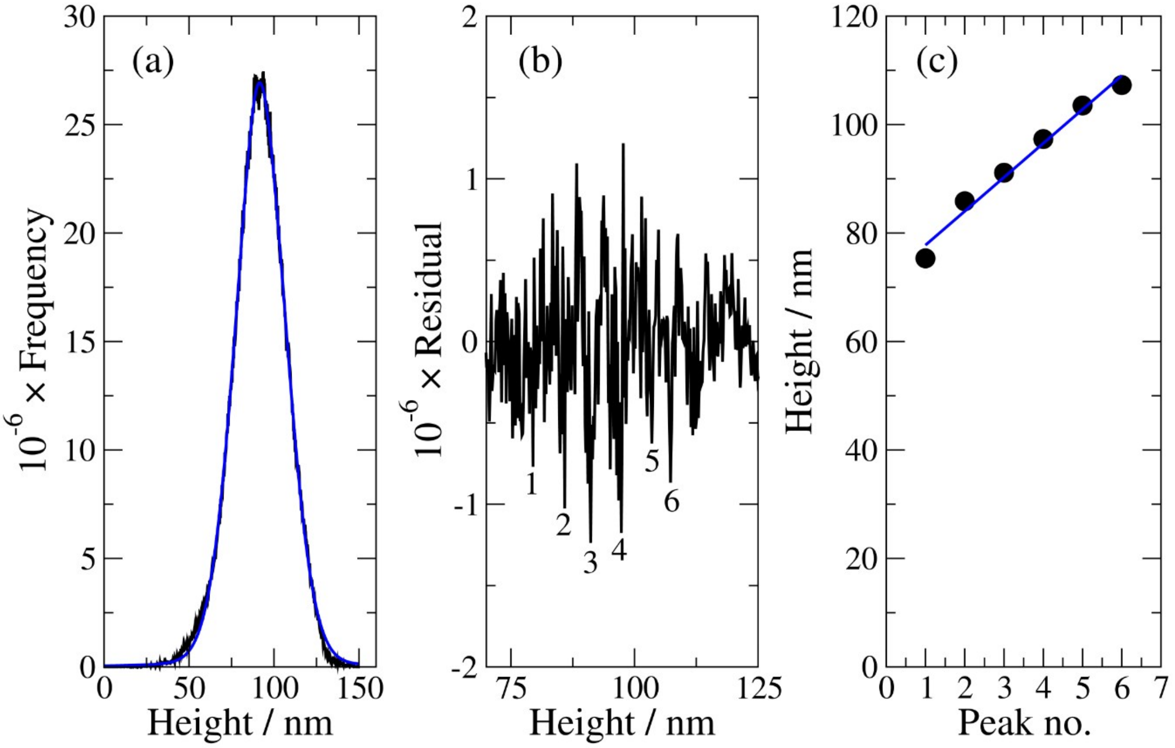

Figure S11. (a) Distribution of height, (b) A fitted pseudo-Voigt function of the measured distribution by a standard least-square method and the residual and (c) Estimated fibre diameter (6.3 +/- 0.45 nm).

References

1. David Nečas and Petr Klapetek, *Central European Journal of Physics*, 2012, **10**, 181–188

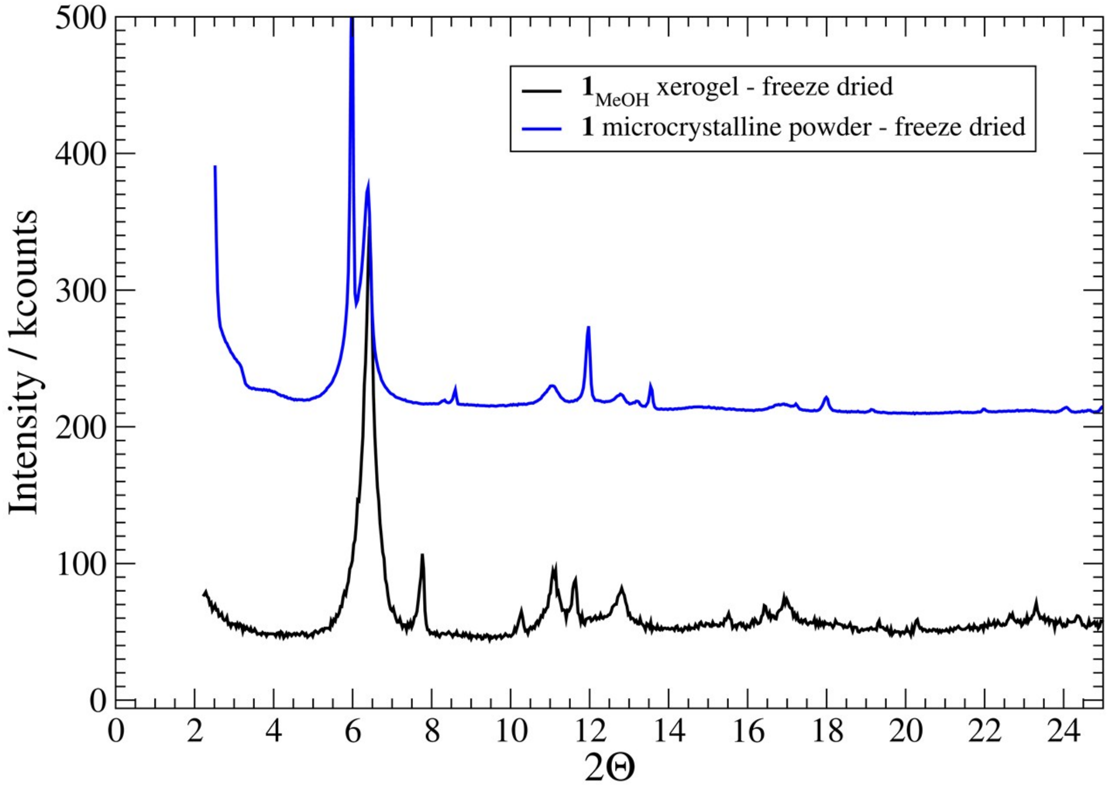

Figure S12. XRD data for freeze-dried samples of xerogel **Xe1**, (lower) and **1** (upper).

Conductivity Measurements, IV and EFM.

Electrical measurements (I-V). Current/voltage curves were recorded on the probe station (Cascade Microtech with a B1500A parameter analyser, Agilent). All of the electrical measurements were carried out under dry nitrogen without light illumination. Platinum MBEs (Smart Microsystems Pt MB-4000, Windsor Scientific Ltd., Slough, UK) were used to fabricate electronic devices for electrical characterisation of **Xe1**. The MBEs were made on Si/SiO_2 substrates. Four independent platinum electrodes were patterned on the top of the SiO_2 layer. The height of the electrodes is 200 nm and their width is 10 µm with 10 µm spaces between them. The surfaces of the MBEs were electrically insulated except for a 2 × 2 mm^2 area for depositing the gel. The platinum MBEs were washed with ethanol and dried with nitrogen gas. The clean platinum electrodes were analysed on a probe station and reference current/voltage curves were recorded, which showed the background currents to be less than 100 fA at 2 V. In the case of the xerogel a drop (0.5 µl) of $\mathbf{1}_{MeOH}$ was cast onto the platinum MBEs and the sample was kept in a closed vial to allow the self-healing of the gel and then left to dry. The gel droplet dried to produce a film of the **Xe1** fibres across the Pt-electrodes. The electrodes were connected to the probe station and current/voltage curves were collected.
For single crystal measurements, a crystal of **1** was electrically insulated on a glass slide and the crystal were connected either directly to probe needles or the ends of the crystal were coated with Ga-In eutectic and probe needles were attached to these pads. In both cases the

probe needles were positioned at opposite ends of the long crystal axis. Both methods for contacting the crystal gave essentially similar results (see Figure S13a and S13b).

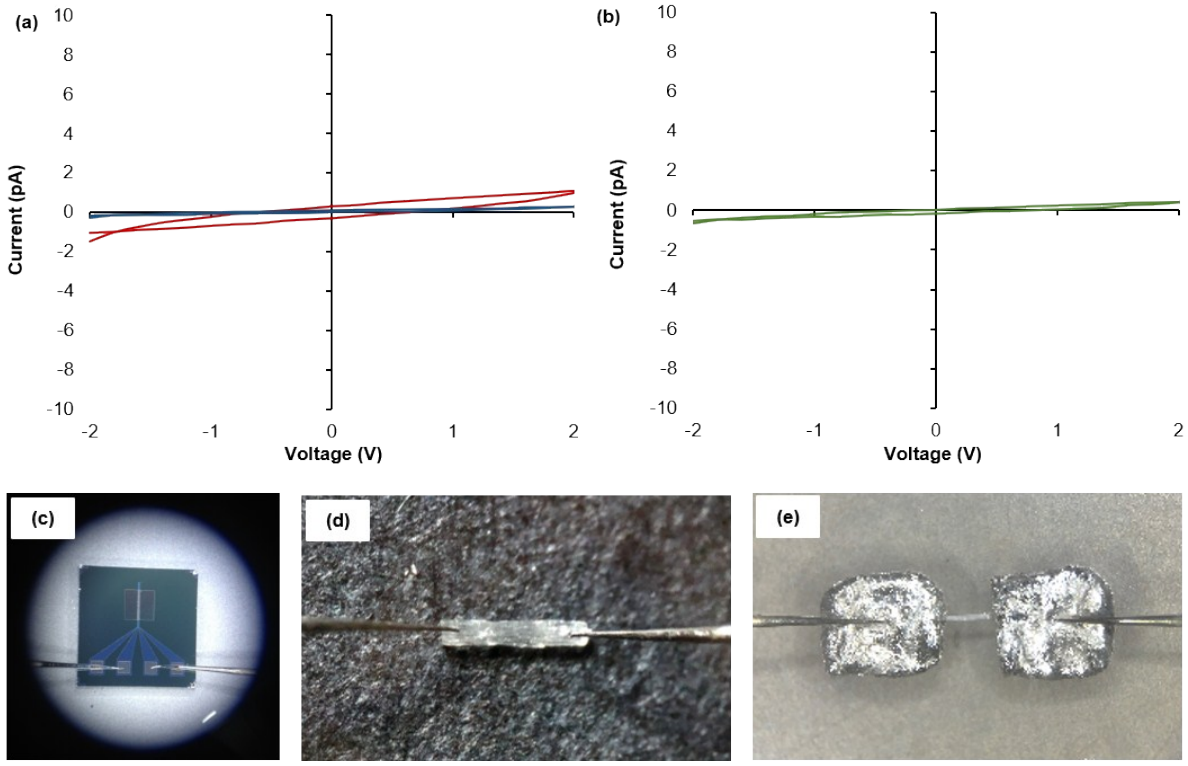

Figure S13. I-V curves of (a) the **Xe1** (red) and an oriented single crystal of **1** directly contacted to probe needles (blue), and (b) data for an oriented single crystal of **1** contacted to probe station tips through Ga-In eutectic (green) as shown in (e). Optical images of (c) microband electrodes used for I-V measurements of xerogel **Xe1**, (d) a single crystal of **1** directly contacted to probe needles (blue) and (e) a single crystal of **1** contacted to probe needles through Ga-In eutectic (green).

Electrostatic Force Microscopy (EFM). Electrostatic force microscopy (EFM) operated in the dynamic non-contact mode provides a useful contactless tool for qualitative testing of the conductivity of a single nanowire prior to any quantitative measurement of its conductivity.[1,2] The method is based on the storage of energy in the tip/nanowire/substrate capacitor and the nanowire conductance influences the measurement *via* the RC time constant for polarisation of the nanowire. For EFM studies, the nanowires are aligned on Si/SiO_2 chips with an oxide thickness ~200 nm and the phase shift between the driving force and the tip motion is recorded as a function of applied dc bias (V) as the tip crosses above the fibre at a constant lift height. As shown by Staii and co-workers,[3] a negative phase shift with a V^2 applied bias dependence is an indication of a conductive nanowire. Objects which are merely polarisable and do not have charges which can move away from the vicinity of the tip produce a positive phase shift; this conclusion is independent of the details of the quantitative model of the effect.

Both EFM phase images and the corresponding topographic images were acquired using the Multimode 8 atomic force microscope (Bruker, Germany). Data were analysed using NanoScope Analysis 1.5 software. EFM was performed in air using AFM tips on n-doped Si cantilevers (MESP-LM-V2, Bruker), both of the probe sides were coated with Cobalt/Chromium layer. The nominal tip radius was approximately 25 nm, resonant frequency 75 kHz, and spring constant k ~3 N/m. The EFM phase images has been captured at applied DC bias between +10 and -10 volts with a scan lift height of 30 nm. The sample of $\mathbf{1}_{MeOH}$ gel was prepared as above and diluted 20 times with further methanol. The sample for analysis

was prepared by drop-casting 1 µL of the diluted cytidine-Ag(I) solution onto a Si<100>/200 nm SiO_2 slide. The Si slides were sonicated in methanol for 15 min, dried with nitrogen gas and then exposed to oxygen plasma (Femto - diener electronic, Plasma-Surface-Technology) for 20 min at 80 W. The slides were washed again with methanol and dried with nitrogen gas. The slide with the drop-cast gel was surrounded by 10 µl of methanol in a closed glass vial (with a volume ~ 9 ml) for 30 min. Then, it was left to dry in air for 10 min.

A control experiment has been carried out to confirm that the fibres in the AFM and EFM images do not arise from any solvent impurity. A Si<100>/200 nm SiO_2 slide was sonicated gently in a sonication bath in methanol for 15 min, dried with nitrogen gas and then exposed to oxygen plasma for 20 min at 80 W. The slides were washed again with methanol and dried with nitrogen gas. A drop of methanol (2 µl) was drop-casted onto the Si/SiO_2 slide and left to dry in air. The sample was scanned by AFM at different locations. The AFM images on this sample have showed no fibres on the surface of the Si/SiO_2.

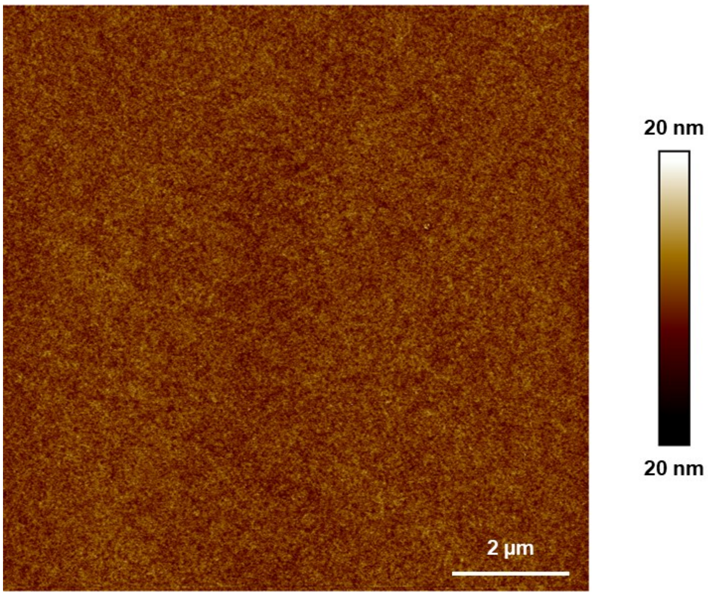

Figure S14. AFM image of a control sample that has been prepared by drop-casting 2 µl of methanol onto a Si<100>/200 nm SiO_2 slide highlighting the absence of fibres from solvent.

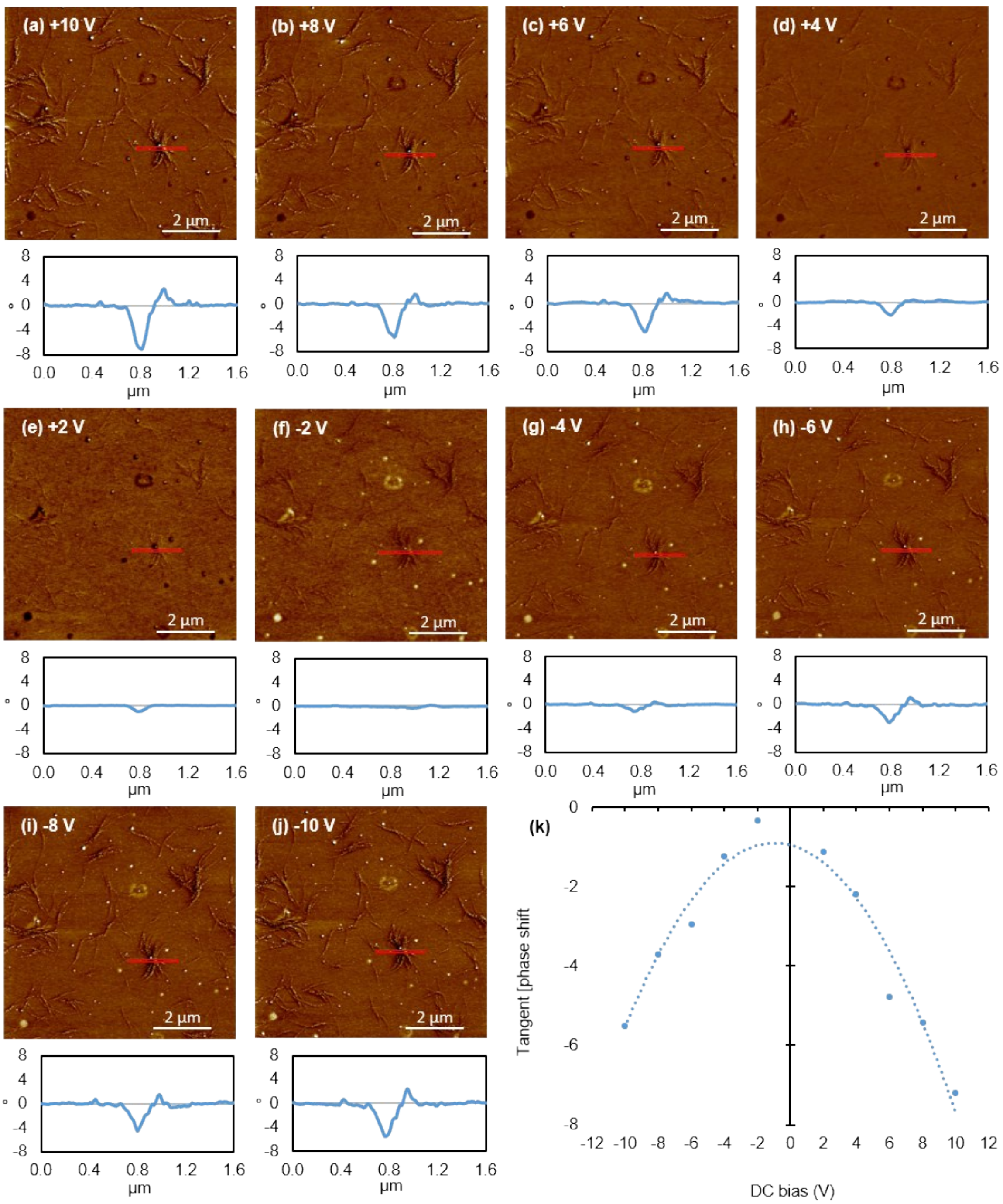

Figure S15. EFM phase image of the **Xe1** and the line profiles corresponding to the boxes in the EFM phase images (a-j). The EFM phase images were captured at applied DC bias between +10 V and -10 V, and a lift height of 30 nm. (k) A plot of the tangent of the measured phase angle arising from the aggregation of fibres as a function of applying different DC biases.

(1) S. M. D. Watson, A. R. Pike, J. Pate, A. Houlton and B. R. Horrocks, *Nanoscale*, 2014, **6**, 4027 – 4037.
(2) M. Bockrath, N. Markovic, A. Shepard, M. Tinkham, L. Gurevich, L. P. Kouwenhoven, M. W. Wu and L. L. Sohn, *Nano Lett.*, 2002, **2**, 187 - 190.
(3) C. Staii, A. T. Johnson and N. J. Pinto, *Nano Lett.*, 2004, **4**, 859 - 862.

Reduction Behaviour

Photoreduction was achieved by suspending vials of **1**$_{MeOH}$ (2 ml) within a Southern New England Ultraviolet Company Photochemical reactor, using eight RMR-3000 (50/60 Hz, 120 Volts, 300 nm) UV bulbs. Exposure times ranged from 1 - 90 minutes.

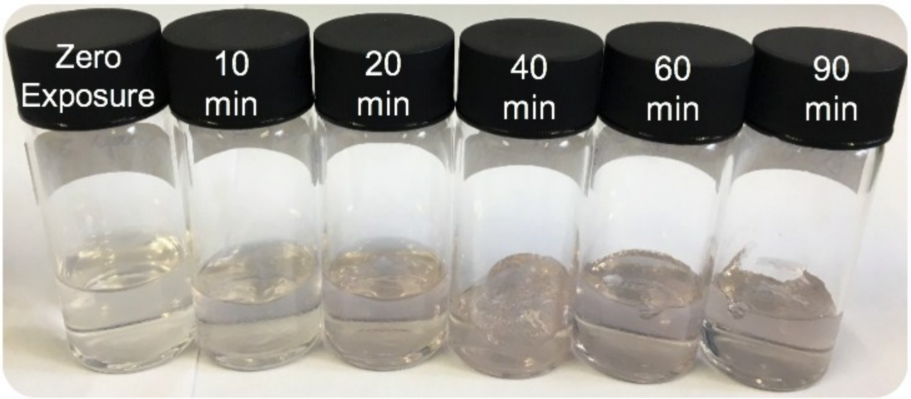

Figure S16. An image showing a series of **1**$_{MeOH}$ samples exposed to UV-light (300 nm) for up to 90 minutes.

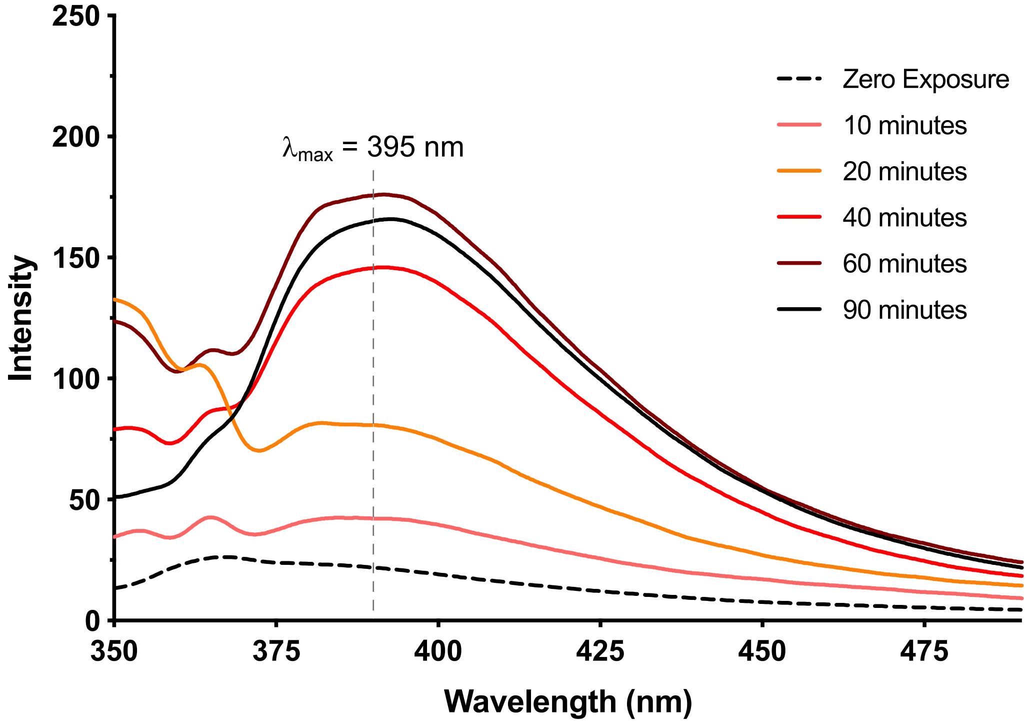

Figure S17. Emission data of **1**$_{MeOH}$ from λ_{ex} of 330 nm.

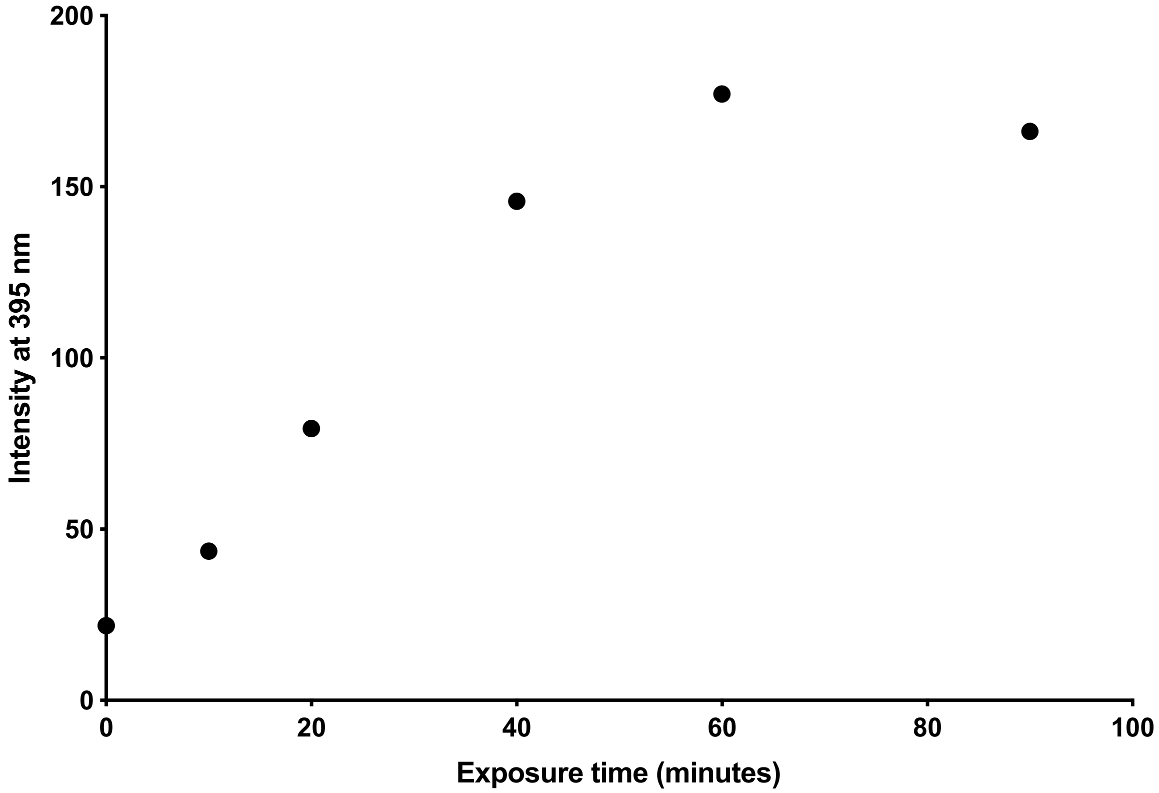

Figure S18. Emission intensity (λ_{em}: 395 nm, λ_{ex}: 330 nm) of photoreduced $\mathbf{1}_{MeOH}$ *vs.* exposure time to UV-light.

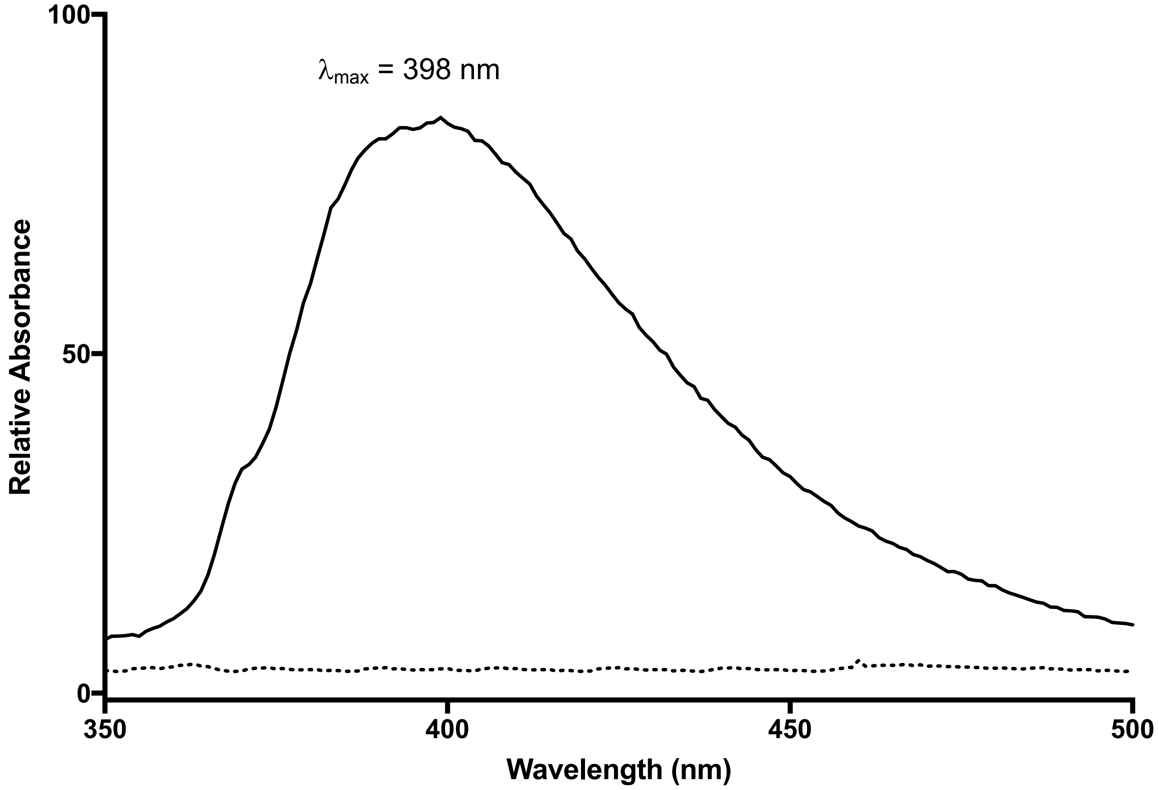

Figure S19. Absorption spectrum for $\mathbf{1}_{MeOH}$ gel after photoreduction (90 minutes) (Solid line) *vs.* zero exposure (dotted line) showing the appearance of the plasmon band for Ag nano-particles.

Samples of $\mathbf{1}_{MeOH}$ gel were drop cast on to a silicon (SiMPore, SN100-A50Q10) grid and then air dried over 15 minutes. The grids are examined using a JEOL 2100F (FEG) Transmission Electron Microscope and digital images are collected using a Gatan Orius CCD camera housed within the School of Physics, Durham University – Dr B. Mendis.

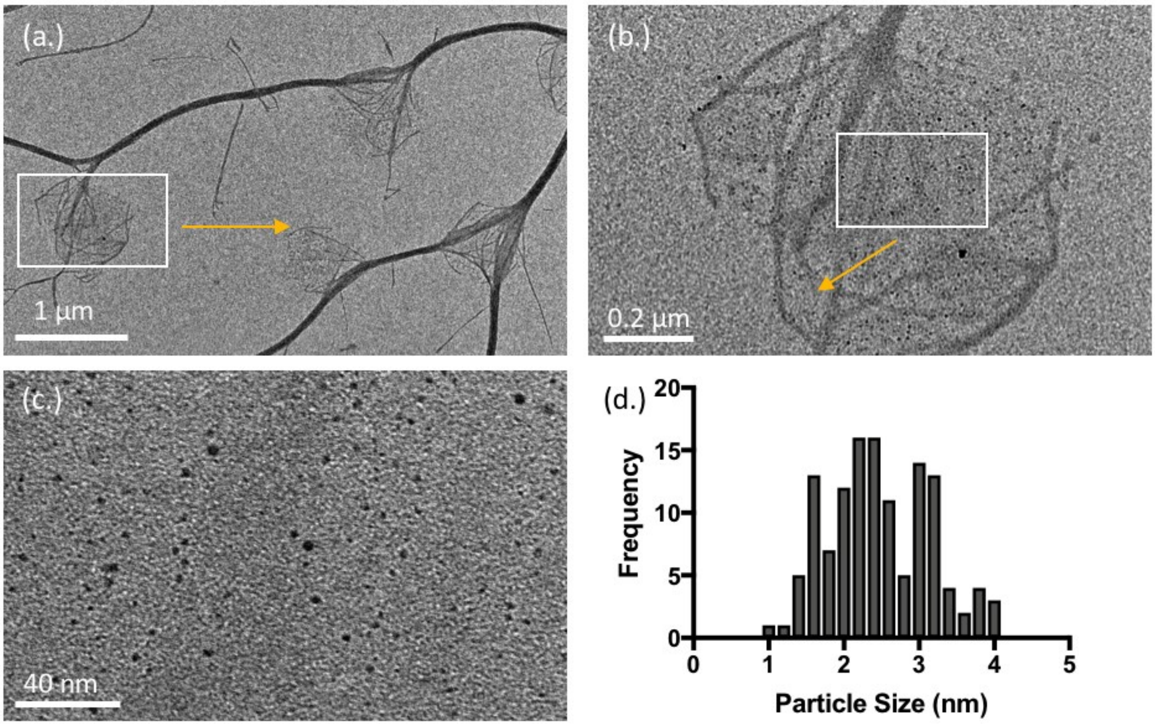

Figure S20. TEM Images of $\mathbf{1}_{MeOH}$ in a xerogel state deposited on a SiN grid; (a. & b.) Bright field images of the xerogel showing long fibres > 5 μm in length (14 – 82 nm width), (c. & d.) Electron dense particles within a fray with particle size distribution analysis (1.0 – 4.0 nm dia., 2.4 nm average dia.

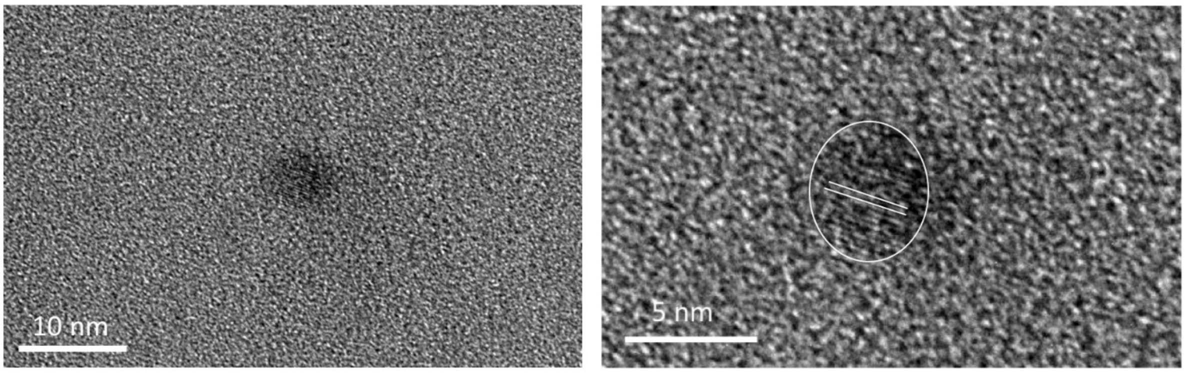

Figure S21. HR-TEM image of a silver particle (4.6 nm dia.) with lattice fringe spacing of 0.25 nm ±0.02 that corresponds to the interplanar distances of Ag(111)

Band Structure Calculations

All spin-polarized density functional theory (DFT) calculations were performed with the Vienna ab-initio Simulation Package (VASP)[1] and the projector augmented wave (PAW) method.[2,3] The exchange-correlation potential was described by the Perdew-Burke-Ernzerhof (PBE) functional[4] extended to incorporate a Van der Waals correction.[5] We use a k-point gird of 1 × 1 × 5, so that there are 5 *k*-points in the transport direction.

1. G. Kresse and J. Hafner, *Phys. Rev. B*, 1993, **47**, 558; 1994, **49**, 14251; G. Kresse and J. Furthmüller, *Phys. Rev. B*, 1996, **54**, 11169; *Comput. Mat. Sci.*, 1996, **6**, 15 (1996).
2. P. E. Blöchl, *Phys. Rev. B*, 1994, **50**, 17953.
3. G. Kresse and D. Joubert, *Phys. Rev. B*, 1999, **59**, 1758.
4. J. P. Perdew, K. Burke, and M. Ernzerhof, *Phys. Rev. Lett.*, 1996, **77**, 3865.
5. S. Grimme, *J. Comput. Chem.*, 2006, **27**, 1787.
